# Automatic Segmentation of the Dorsal Claustrum in Humans Using in vivo High-Resolution MRI

**DOI:** 10.1093/texcom/tgaa062

**Published:** 2020-09-01

**Authors:** Shai Berman, Roey Schurr, Gal Atlan, Ami Citri, Aviv A Mezer

**Affiliations:** Edmond and Lily Safra Center for Brain Sciences, Hebrew University of Jerusalem, Jerusalem 91904, Israel; Edmond and Lily Safra Center for Brain Sciences, Hebrew University of Jerusalem, Jerusalem 91904, Israel; Edmond and Lily Safra Center for Brain Sciences, Hebrew University of Jerusalem, Jerusalem 91904, Israel; Edmond and Lily Safra Center for Brain Sciences, Hebrew University of Jerusalem, Jerusalem 91904, Israel; Department of Biological Chemistry, Institute of Life Sciences, Edmond J. Safra Campus, Hebrew University of Jerusalem, Jerusalem 91904, Israel; Program in Child and Brain Development, Canadian Institute for Advanced Research, Toronto, ON M5G 1M1, Canada; Edmond and Lily Safra Center for Brain Sciences, Hebrew University of Jerusalem, Jerusalem 91904, Israel

**Keywords:** connectome, covariance, insula, subcortical structures

## Abstract

The claustrum is a thin sheet of neurons enclosed by white matter and situated between the insula and the putamen. It is highly interconnected with sensory, frontal, and subcortical regions. The deep location of the claustrum, with its fine structure, has limited the degree to which it could be studied in vivo. Particularly in humans, identifying the claustrum using magnetic resonance imaging (MRI) is extremely challenging, even manually. Therefore, automatic segmentation of the claustrum is an invaluable step toward enabling extensive and reproducible research of the anatomy and function of the human claustrum. In this study, we developed an automatic algorithm for segmenting the human dorsal claustrum in vivo using high-resolution MRI. Using this algorithm, we segmented the dorsal claustrum bilaterally in 1068 subjects of the Human Connectome Project Young Adult dataset, a publicly available high-resolution MRI dataset. We found good agreement between the automatic and manual segmentations performed by 2 observers in 10 subjects. We demonstrate the use of the segmentation in analyzing the covariation of the dorsal claustrum with other brain regions, in terms of macro- and microstructure. We identified several covariance networks associated with the dorsal claustrum. We provide an online repository of 1068 bilateral dorsal claustrum segmentations.

## Introduction

The claustrum is a thin sheet of neurons enclosed between the insular cortex and the putamen. It exists in almost all mammals, and its anatomy has been widely studied across species (Kowiański et al. 1999). Ex vivo studies have found conserved anatomical characteristics across species, such as the existence of claustral puddles, in which the thin claustrum extends to form a thicker region (Johnson et al. 2014).

The deep location of the claustrum, together with its fine structure, has limited the degree to which the region could be studied, particularly in vivo in humans. Nevertheless, several theories attempt to explain the claustrum’s role. Crick and Koch postulated that the claustrum is key for multimodal integration, famously suggesting that the claustrum forms “the seat of consciousness” (Crick and Koch 2005). This hypothesis agrees with recent studies that showed that the claustrum is highly interconnected with other brain regions (Mathur 2014; Goll et al. 2015). Due to these findings, it was suggested that the claustrum acts as a regulator of cortical excitability, and therefore likely plays a crucial role in sensory perception and selective attention (Mathur 2014; Goll et al. 2015). In agreement with these hypotheses, studies in mice found that the claustrum can induce cortical suppression and that silencing the claustrum renders mice more susceptible to distraction (Atlan et al. 2018; Jackson et al. 2018). This small handful of studies still leaves ample room for uncovering the mysterious nature of the claustrum. Its potential role in high cognitive functions, as well as in pathological states (Morys et al. 1996; Dickstein et al. 2006; Kalaitzakis 2014), makes the claustrum a prominent candidate for in vivo studies in humans.

Despite its intriguing nature, the human claustrum has been investigated in vivo in only a handful of neuroimaging studies (Fernández-Miranda et al. 2008; Koubeissi et al. 2014; Milardi et al. 2015; Torgerson et al. 2015; Smith et al. 2017; Krimmel et al. 2019). In part, this can be attributed to the challenge in segmentation of the claustrum, and the lack of an established automatic segmentation tool. Instead, existing studies have used manual segmentation, which is very time-consuming. Ongoing advances in magnetic resonance imaging (MRI) now allow for the acquisition of human brain images with submillimeter resolution, in which the segmentation of the claustrum may be feasible. Establishing a method for automatic segmentation of the claustrum in vivo is therefore a substantial step toward facilitating reproducible analysis of the anatomy, function, and connectivity of the claustrum in humans.

**Figure 1:**
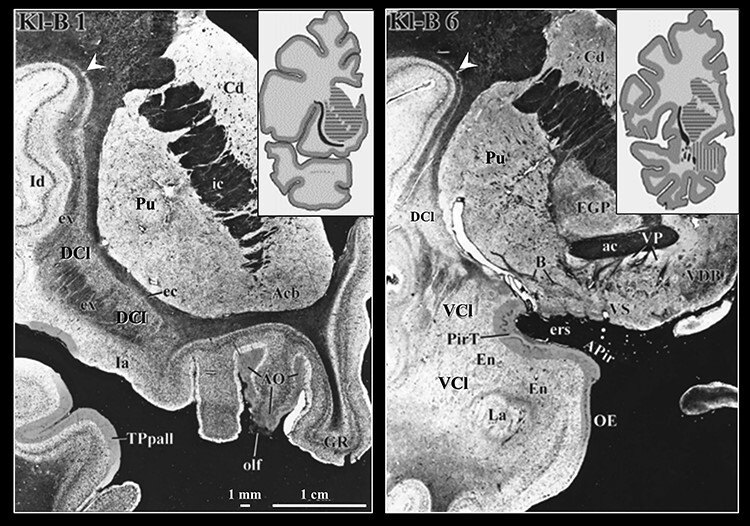
Coronal sections of a human brain stained for myelin and cell bodies with the Klüver–Barrera method. Left: The dorsal claustrum is situated between the putamen and the insular cortex. Its anterior dorsal aspect is a thin layer of gray matter (<1 mm) that bends laterally over the central insular sulcus (white arrowhead). Right: The more fragmented ventral claustrum appears inferior to the putamen and is harder to distinguish from its surroundings. Insets: the approximate location of each section. Black: claustrum, horizontal stripes: putamen and caudate, vertical stripes: amygdala. Main slices modified with permission from Sakamoto et al. (1999). Insets reproduced with permission from Crick and Koch (2005). DCl: dorsal claustrum, ex: extreme capsule, Id: dorsal insular cortex, Pu: putamen, VCl: ventral claustrum.

Figure 1 demonstrates key aspects of the claustrum anatomy in post mortem sections (Heimer et al. 1999). These anatomical features are relevant for the segmentation approach we propose in this work. The claustrum comprises of 2 distinct structures: a dorsal claustrum and a ventral claustrum (Morys et al. 1996; Kowiański et al. 1999; Fernández-Miranda et al. 2008). The dorsal claustrum is more compact and has higher cell density compared with the ventral claustrum (Rae 1954; Kowiański et al. 1999). In terms of morphology, in its anterior part, the dorsal aspect of the dorsal claustrum appears as a thin layer of gray matter (<1 mm) that bends laterally over the central insular sulcus (Morel et al. 2013). The dorsal claustrum widens ventrally, where it is situated between the putamen and the insular cortex, following the curvature of the putamen. Occasionally, the claustrum widens and extends toward the insular cortex to form “puddles.” Finally, at its most inferior-anterior part, it bends beneath the putamen. The transition from dorsal to ventral claustrum occurs where the dorsal claustrum bends medially (Fig. 1*B*; Davis 2008). At this level, the ventral and dorsal claustra often appear disconnected (Rae 1954; Davis 2008). The ventral claustrum, also termed the fragmented claustrum, consists of scattered islands of gray matter in between fibers of the white matter (Druga 2014). At typical resolutions of in vivo MRI, this fragmented nature manifests as partial volume effects with the surrounding white matter. Hence, the ventral claustrum is not always apparent in MRI images (Davis 2008). Given the limitations of reliably identifying the ventral claustrum, this work focuses on segmenting the dorsal claustrum.

Knowledge regarding the connectivity of the claustrum arises primarily from animal studies. In these studies, the claustrum has been shown to be a highly connected region (e.g., Olson and Graybiel 1980; Sadowski et al. 1997; Edelstein and Denaro 2004). Tracing studies in mice showed that the claustrum is connected with frontal and sensory brain regions (Smith and Alloway 2014; Atlan et al. 2017; Wang et al. 2017). In rats, a combined tracing and resting state functional MRI (rs-fMRI) study found that the claustrum is functionally connected to its contralateral counterpart and is anatomically connected with the contralateral hemisphere, as well as regions that include the cingulate cortex, the agranular motor cortex, and the medial prefrontal cortex (Smith et al. 2017). One tracing study that focused on the prefrontal cortex found claustral connections with the prefrontal cortex in nonhuman primates as well (Reser et al. 2014).

Since axonal tracing is impossible in humans, the tool of choice for studying the connectivity of the claustrum in humans is MRI. One approach for studying functional connectivity is to measure correlations of blood-oxygenation level-dependent signal in fMRI. Using this approach, Krimmel et al. (2019) found that at rest, the claustrum was associated with cortical regions such as the anterior cingulate, prefrontal cortex, and parietal cortex. During tasks, the same study found that claustrum activity was associated with widespread cortical activation. Another approach used to study the connectivity of the claustrum in vivo is diffusion MRI (dMRI) tractography. This approach also yielded connections between the human dorsal claustrum and multiple cortical regions, including the frontal lobe and cingulate cortex (Torgerson et al. 2015). Importantly, given the spatial resolution of fMRI and dMRI (commonly ~ 2 mm^3^), it is inherently hard to separate the claustrum—which is less than ~ 1 mm thick in some regions—from the neighboring putamen and insula. Indeed, existing studies often refer to “claustrum/insula” activations due to this limitation (Fassbender et al. 2011; Ersche et al. 2012; Wang et al. 2019; Snider et al. 2020).

A third approach closely related to brain connectivity is structural covariance analysis (Andrews et al. 1997; Mechelli et al. 2005; Wei et al. 2018). Structural covariance describes the phenomenon in which the morphology or microstructure of 2 or more brain regions covaries across subjects (Alexander-Bloch, Giedd, et al. 2013). Structural covariance is often based on the correlation of macrostructural properties such as the volume or thickness of brain regions. The resulting set of covarying regions is termed a covariance network. Studies have shown that these networks converge, to a certain extent, with networks derived from other modalities: networks of dMRI tractography (Gong et al. 2012), maturation-related networks (Alexander-Bloch, Raznahan, et al. 2013; DuPre and Spreng 2017), resting state fMRI networks (Kelly et al. 2012), and transcriptomics brain networks (Romero-Garcia et al. 2018). Recently, additional structural quantitative MRI measurements were used in a structural covariance analysis, revealing networks that may be related to amyloid deposition (Ye et al. 2019). The biological source of these converging results remains an ongoing discussion, and covariance is not a direct measure of connectivity. Nevertheless, several mechanisms have been suggested to explain structural covariance, including physical connectivity of white matter tracts between regions, as well as synchronous co-activation, co-regulation, or co-development of regions (Mechelli et al. 2005; Alexander-Bloch, Giedd, et al. 2013). As a proxy for brain connectivity, structural covariance provides insight into the set of brain regions that possibly subserve a similar function. Structural covariance networks are especially useful in studying fine structures like the claustrum, in which the larger voxels required by dMRI and fMRI connectivity analyses lead to greater partial volume effects. Hence, in this study, we used the structural covariance analysis. This approach has never been applied to study the association of the claustrum with other brain regions, probably because it requires the identification of the claustrum in a large cohort of subjects, which is impractical using manual segmentation.

In this study, we developed a fully automatic algorithm for segmenting the human dorsal claustrum in vivo using high-resolution T1-weighted (T1w) images. Specifically, we used the Human Connectome Project (HCP) Young Adult dataset, a publicly available, high-quality, high-resolution dataset (Van Essen et al. 2013). We used this algorithm to segment the bilateral claustrum in 1068 subjects. To assess the validity of our algorithm, we manually segmented the claustrum in 10 subjects and compared the manual results to the automatic results. We compared the automatic segmentation to manual segmentations in a subset of subjects. The automated segmentation supported the application of structural covariance analysis, an approach that serves as a proxy for investigating the structural brain connectivity based on micro- and macrostructural properties.

We make the segmentation code, as well as all segmentation results publicly available at https://github.com/MezerLab/ClaustrumSegmentation and https://doi.org/10.5281/zenodo.3960552, respectively. Our work will enable future investigation of the claustrum, including cognitive behaviors such as multimodal tasks of selective attention.

## Materials and Methods

### Subjects and Data

To develop a method for the automatic segmentation of the claustrum, we used the publicly released dataset of the HCP (Van Essen et al. 2013). The HCP Young Adults dataset consists of 1206 healthy subjects acquired on a 3T connectome scanner. The data includes high-resolution (0.7 mm^3^ isotropic) T1-weighted (T1w), and T2-weighted (T2w) structural images and multishell high angular resolution diffusion imaging scans (1.25 mm^3^ isotropic, 90 diffusion directions, b = 2000 mm/s^2^). The downloaded diffusion data had been processed using the HCP preprocessing pipeline (Glasser et al. 2013). In short, the preprocessing steps include intensity normalization across runs, FSL’s TOPUP algorithm for echo planar imaging (EPI) distortion correction (Andersson et al. 2003; Andersson and Sotiropoulos 2016), FSL’s EDDY algorithm for eddy current and motion correction, gradient nonlinearity correction and the registration of mean b0 to native volume T1w.

The subjects of this dataset are healthy young adults. HCP exclusion criteria were neurodevelopmental disorders, documented neuropsychiatric disorders, neurologic disorders, diabetes, or high blood pressure. Of the 1206 subjects, we chose the 1113 subjects who have structural MRI data. We further removed 35 subjects whose data were problematic for FreeSurfer segmentation and surface reconstruction (“Issue code B”; see https://wiki.humanconnectome.org/pages/viewpage.action?pageId=88901591), and nine subjects for whom the putamen was not segmented properly using FSL’s FIRST (Jenkinson and Smith 2001; Jenkinson et al. 2002). The remaining 1069 subjects were included in the present study.

Of the remaining subjects that had diffusion data, we removed 6 subjects with bad diffusion data (missing slices, negative mean diffusivity (MD) values, or extremely high MD values), leaving 1014 subjects with diffusion data. Analyses were performed with the maximum number of available data for each analysis. We report the respective available number of subjects for each analysis. For further information and details of data acquisition and processing in this sample, please see: https://www.humanconnectome.org/study/hcp-young-adult/document/1200-subjects-data-release.

### Claustrum Segmentation

#### Automatic Segmentation

**Figure 2:**
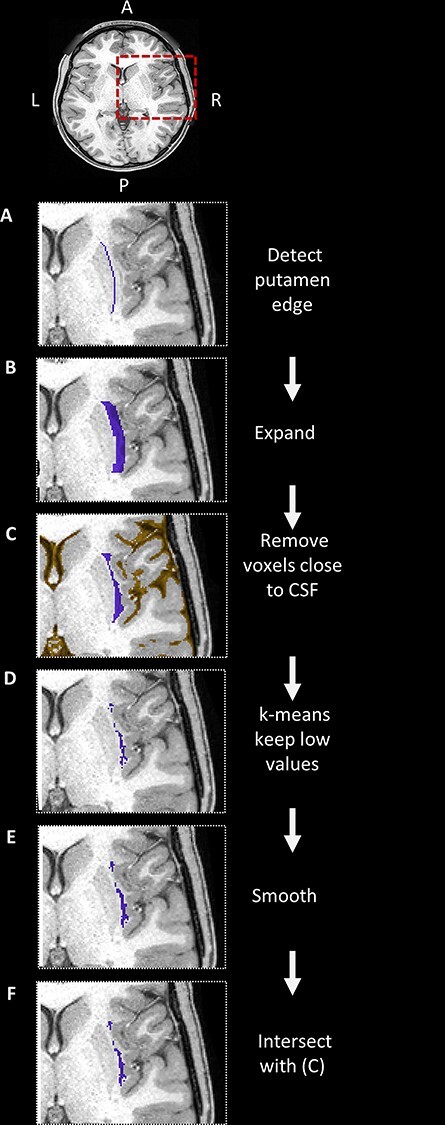
Automatic segmentation of the claustrum. The steps of the segmentation algorithm are presented for the right hemisphere on images obtained from 1 example subject. The relevant ROIs are overlaid on a T1w image, in the area shown in a red box. (*A*) First, we detect the lateral edge of the putamen. (*B*) We expand the putamen edge laterally. (*C*) We exclude any voxel close to the CSF (orange). (*D*) We cluster the voxels using *k*-means (*k* = 2) and choose the cluster of lower T1w values. (*E*) We smooth the result, and (*F*) take the intersection with the ROI created in step (*C*). For details, see Methods. Key: A: anterior; P: posterior; L: left; R: right.

We developed an automatic procedure for claustrum segmentation (Fig. 2), based on anatomical landmarks. This algorithm capitalizes on the established segmentations of the putamen using FSL’s FIRST (Jenkinson and Smith 2001; Jenkinson et al. 2002), and of the cerebrospinal fluid (CSF) using MRtrix’s function 5ttgen (Smith et al. 2012; Tournier et al. 2019). We begin by automatically defining a general region of interest (ROI) based on the anatomical landmarks surrounding the claustrum. The first step is to detect the lateral edge of the putamen, which is located medially to the claustrum (Fig. 2*A*). Next, we expand this edge laterally toward the insular cortex by 5 mm, to include the entire claustrum (Fig. 2*B*). The resulting ROI may include voxels of the insular cortex, which have similar intensity values as the claustrum, and hence might be erroneously included in the claustrum segmentation. Since available tools for insula segmentation, such as FreeSurfer, are also prone to mistakes in this region, we did not rely on insular segmentation to exclude these voxels. Instead, we remove all voxels within a 5-voxel range from the segmented CSF, which nicely follows the cortical folding of the insula (Fig. 2*C*). After this step, the resulting ROI includes mostly claustrum and white matter voxels. To separate the claustrum voxels from the surrounding white matter, we use the *k*-means clustering algorithm (*k* = 2) on the T1w values. The cluster with the lower values serves as an initial segmentation of the claustrum (Fig. 2*D*). To obtain a continuous representation of the claustrum, we use two-dimensional smoothing along the anterior–posterior and ventral-dorsal axes (Fig. 2*E*). Finally, due to the curved shape of the claustrum, this smoothing may introduce unwanted voxels into the segmentation. We therefore restrict the claustrum segmentation to the initial ROI (Fig. 2*E*). The MATLAB code for claustrum segmentation is available at https://github.com/MezerLab/ClaustrumSegmentation.

#### BrainSuite’s Automatic Segmentation

BrainSuite is a tool for cortical parcellation and subcortical segmentation, which is based on surface-constrained volumetric registration of individual MRI images to a manually labeled atlas (Joshi et al. 2007). The BrainSuiteAtlas1 atlas, which is based on a high-resolution T1w image (0.5 × 0.5 × 0.8 mm), includes the claustrum. To compare the claustrum segmentation of BrainSuite with our proposed segmentation, we used the BrainSuite software (version 18a^3^; http://brainsuite.org) in the same 10 subjects we used for manual segmentation.

#### Manual Segmentation

To validate our automatic segmentation, we manually segmented the claustrum bilaterally in 10 randomly selected subjects using the T1w image. Our approach for manual segmentation was similar to that described in detail by Davis (2008). Each subject was segmented twice, by 2 different observers (S.B. and R.S.). These manual segmentations were used as ground truth to evaluate the performance of the automatic segmentation.

### Segmentation Evaluation

To evaluate the performance of the automatic claustrum segmentation, we used the Dice similarity coefficient (Dice 1945):

$$ \mathrm{Dice}\ \mathrm{coefficient}=\frac{2\left(A\cap B\right)}{A+B}, $$

where *A* is the set of voxels of 1 segmentation, *B* is the set of voxels of the other segmentation, and $A\cap B$ are the voxels shared by both segmentations. Specifically, we compared the correspondence between the 2 manual segmentations, as well as between the manual segmentation and each of the 2 automatic methods.

### Structural Measurements of the Claustrum

To characterize the claustrum’s macro- and microstructure, we calculated 3 properties of the segmented claustrum: its volume, its mean T1w/T2w values, and its mean MD.

- Volume: The volume of each claustrum was calculated as the number of voxels in the segmentation, multiplied by the voxel volume of 0.7^3^ mm^3^.
- Mean T1w/T2w: Calculating the ratio between the T1w and T2w images produces a semiquantitative contrast, as it removes most of the shared biases of the images (such as the receive-coil inhomogeneities). The T1w/T2w contrast is sensitive to myelin (Glasser and Van Essen 2011; but see Arshad et al. 2017; Uddin et al. 2018). To control for remaining subject-specific bias, we followed the approach of (Glasser and Van Essen 2011) and calculated for each subject the *z*-score of the T1w/T2w values over all brain regions (see “Structural covariance analysis” below). Thus, the claustrum’s *z*-score of the T1w/T2w values reflects its microstructure relative to the rest of the brain regions.
- MD: Fitting a tensor model to the dMRI data allows the extraction of simple scalar metrics, such as the MD. MD has been related to the tissue architecture, specifically to the amount of membrane in the tissue (Le Bihan et al. 2001). We used CAMINO to fit a tensor to the preprocessed data and extract an MD map for each subject (Hall and Alexander 2009).

### Structural Covariance Analysis

To identify which cortical and subcortical regions are associated with the claustrum in terms of its structural properties, we performed a structural covariation analysis across our subjects. To this end, we parcellated the brain into different regions. Subcortical regions were segmented using FSL’s FIRST, and the mean values (volume, T1w/T2w, and MD) were taken for each ROI. Cortical regions were parcellated by FreeSurfer using the Desikan–Killiany atlas, as provided by the HCP (Desikan et al. 2006). The volume of cortical regions was extracted from the FreeSurfer stats file. The mean T1w/T2w and MD values for each cortical region were calculated using FreeSurfer, which samples the images values within each region along the mid-thickness surface between the pial surface and the white matter surface (Glasser et al. 2013).

Combining the bilateral measurements of 35 cortical regions and 5 subcortical regions (putamen, caudate nucleus, thalamus, hippocampus, and the automatically segmented claustrum) resulted in a vector of values for 80 brain regions (40 from each hemisphere). We used each of the 3 aforementioned parameters separately (volume, T1w/T2w ratio, and MD). Structural correlation analysis is sensitive to subject-specific biases, as these might affect widespread brain regions, and lead to spurious correlations across subject. This is particularly important when dealing with semiquantitative measures like T1w/T2w images or measures that depend on a global parameter like the intracranial volume. Therefore, to account for any biases that may exist between subjects, we calculated the *z*-score of each measurement across the brain regions. Finally, to estimate which region covaries with the claustrum, we calculated the Pearson correlation coefficient across subjects between the standardized values of the claustrum and the standardized values in each of the other brain regions.

## Results

**Figure 3:**
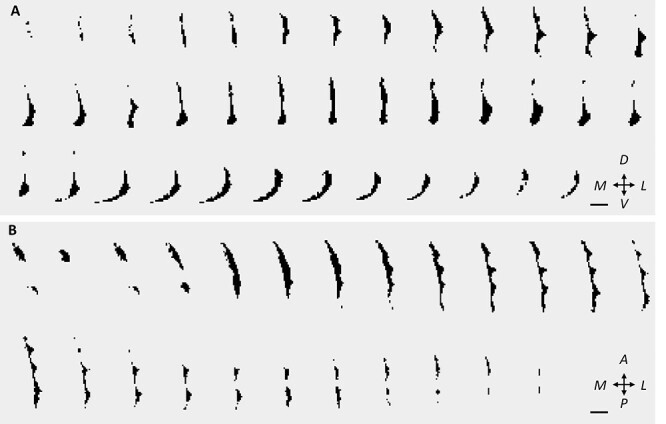
The claustrum in coronal and axial views. Sequential slices of the automatically segmented right claustrum in 1 subject. (*A*) Coronal slices from posterior to anterior. (*B*) Axial slices from ventral to dorsal. Scale bar: 10 mm. See also Supplementary Video 1 for a 3D representation. Key: M: medial, L: lateral; V: ventral; D: dorsal; P: posterior; A: anterior.

We used our automatic algorithm (Fig. 2) to segment the dorsal claustrum bilaterally in 1069 subjects. As expected, the segmentation defines a fine structure of the claustrum, situated between the putamen and the insular cortex. We found that it is narrowest in its dorsal end and grows wider toward its inferior end, at the inferior puddle (Fig. 3*A*, coronal slices). In its most inferior part, the dorsal claustrum curves medially, just below the putamen, in agreement with previous accounts (Rae 1954; Hinova-Palova et al. 2014). Axial slices of the segmented claustrum reveal the characteristic protrusions, or puddles, of the claustrum in the insular gyri (Fig. 3*B*). The resulting segmentation is missing the claustrum’s most anterior–superior aspect that bends over the insula. This small segment is thinner than our voxel size and is outside the ventral-dorsal range of the putamen. Therefore, we could not detect it using the proposed approach. In a single subject, the automatic segmentation did not succeed due to a large blood vessel passing through the claustrum, and that subject was removed from further analyses.

**Figure 4:**
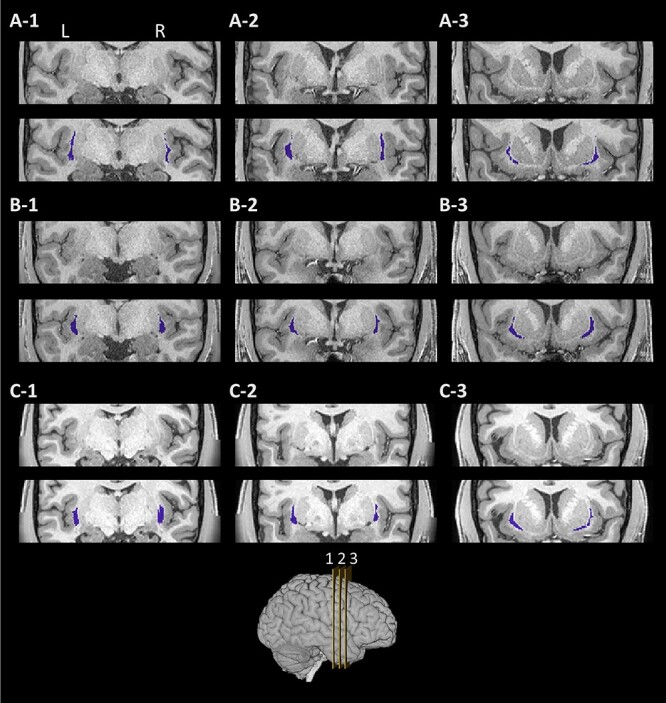
Example segmentations in coronal view. The segmented claustrum in 3 example subjects (rows, *A–C*). For each subject, the region of the claustrum is shown in a T1w image without (top), and with the results of the proposed segmentation (bottom, blue). Each column (1–3) denotes a different coronal slice. Slices 1, 2, and 3 mark 30, 50, and 70% of the putamen length, respectively, from posterior to anterior. Key: L: left; R: right.

**Figure 5:**
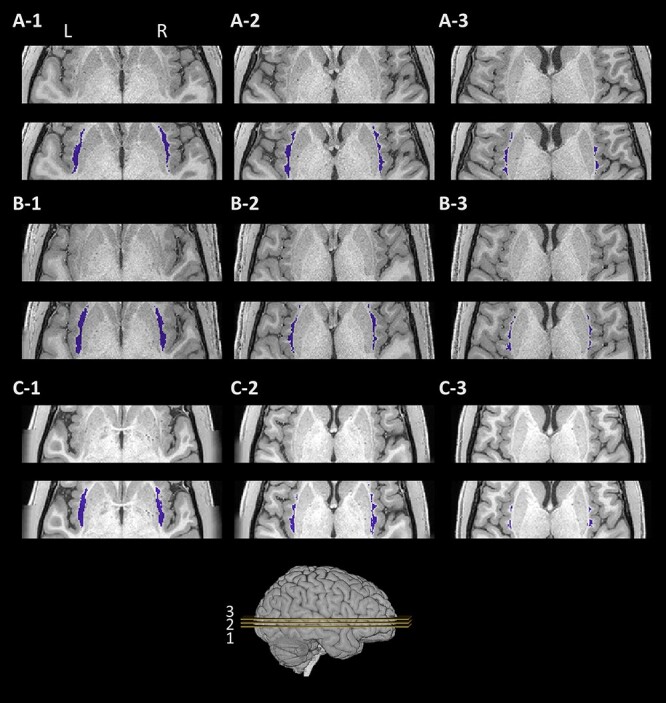
Example segmentations in axial view. The segmented claustrum in the 3 subjects as in Figure 3 (rows, *A–C*). For each subject, the region of the claustrum is shown in a T1w image without (top), and with the results of the proposed segmentation (bottom, blue). Each column (1–3) denotes a different axial slice. Slices were selected at 30, 50, and 70% of the putamen height, from ventral to dorsal. Key: L: left; R: right.

The shape of the dorsal claustrum varies between subjects, though the overall characteristics are maintained. Figures 4 and 5 show the coronal and axial slices, respectively, of the bilateral claustra in 3 subjects, overlaid on their respective T1w image. Notably, parts of the ventral claustrum (not segmented) can be seen in one of these subjects (Fig. 4*C*-1). In Supplementary Video 1, we show a 3-dimensional view of the segmented claustrum in an example subject.

**Figure 6:**
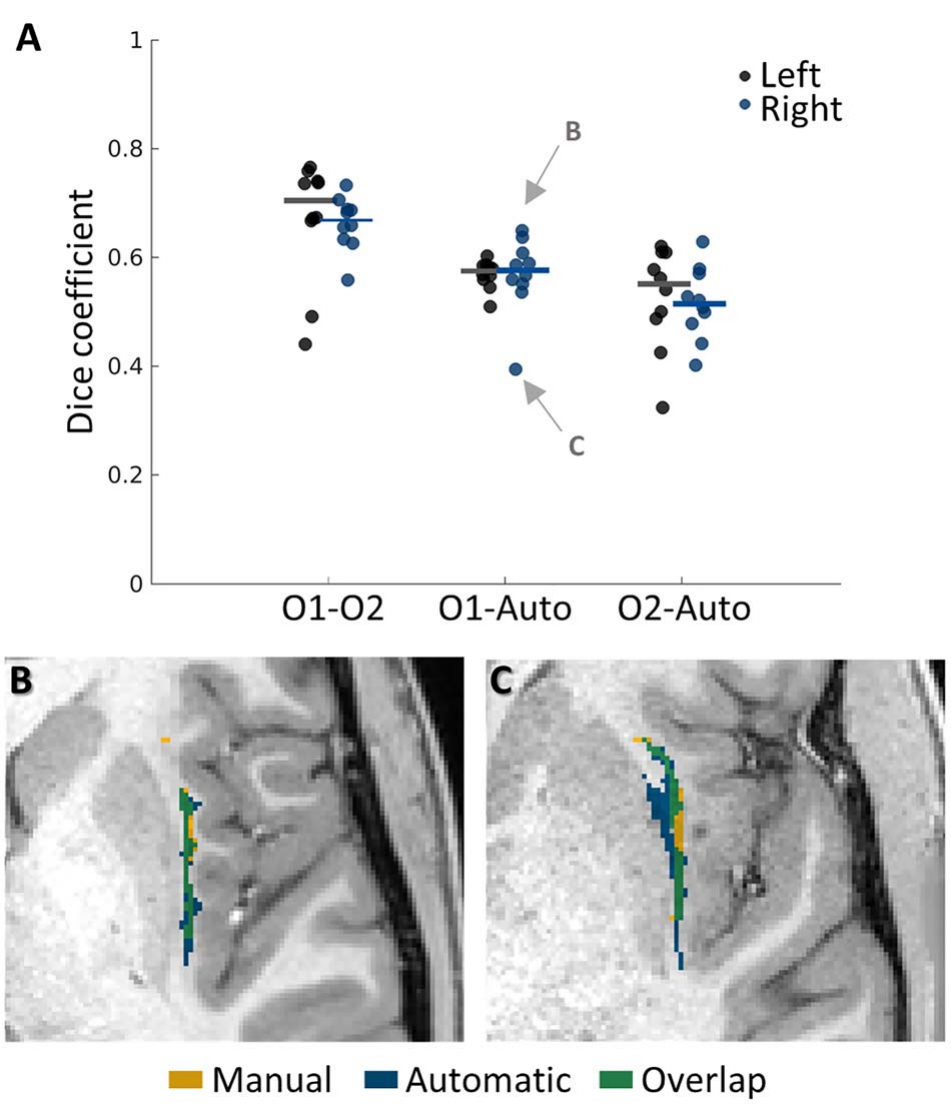
The agreement between manual and automatic segmentation. The bilateral claustra of 10 subjects were manually segmented by 2 observers (O1 = observer 1; O2 = observer 2; auto = automatic segmentation algorithm described herein). (*A*) The agreement between the different segmentations was quantified using the Dice coefficient. On average, the greatest agreement was found between the 2 manual segmentations. The agreement between our automatic segmentation and the 2 observers is moderate, as expected by the small size of the claustrum. Dots represent individual subjects. Lines represent medians. (*B–C*) Axial slices of the right claustrum for the best subject (*B*) and worst subject (*C*) when comparing with observer 1, as marked in panel A. Notice that in (*C*), the segmentation error results mainly from the inaccurate putamen segmentation, so that some of the voxels labeled as associated with the claustrum (in blue) are actually overlapping with the putamen. O1: observer 1 (manual segmentation); O2: observer 2 (manual segmentation).

To assess the quality of the proposed algorithm, we used data from 10 randomly selected subjects. We estimated the agreement between the 2 manual segmentations, and further compared them against the 2 automatic segmentations: our algorithm and BrainSuite’s segmentation (Fig. 6 and Table 1): the proposed automatic segmentation, the automatic segmentation of BrainSuite, and the manual segmentations of 2 observers. As might be expected due to the small size of the claustrum (see Discussion), we observed moderate Dice coefficient values for the comparison of the 2 manual segmentations, with median values of 0.70 and 0.67 for the left and right hemispheres, respectively. The comparison of our automated method with observer 1 gave median values of 0.57 and 0.58, with lower values of 0.55 and 0.51 for observer 2. We found the lowest Dice coefficients between the BrainSuite method and the 2 manual segmentations, with median values of 0.04 and 0.10 for observer 1, and 0.01 and 0.05 for observer 2. Due to the unsuccessful segmentation by BrainSuite, we did not include the BrainSuite segmentations in further analyses. To investigate the source of errors between our automatic segmentation and the manual segmentations, we visually inspected the resulting segmentations. We found that the errors are mostly false negatives, namely that our automatic segmentation includes most of the claustrum, but occasionally some of the surrounding white-matter tissue as well. Together, these results suggest that despite some errors due to partial volume effects, our automatic algorithm captures the structure of the dorsal claustrum with high sensitivity.

**Table 1:** Comparison between segmentation methods. Summary statistics of median (mdn) minimum, (min) and maximum (max) values of the Dice coefficient calculated for pairs of claustrum segmentation methods, across 10 subjects. Key: L: left; R: right; O1: observer 1 (manual segmentation); O2: observer 2 (manual segmentation)

	Interobserver agreement		Automatic segmentation				BrainSuite segmentation			
	O1 with O2		with O1		with O2		with O1		with O2	
	L	R	L	R	L	R	L	R	L	R
mdn	0.70	0.67	0.57	0.58	0.55	0.51	0.04	0.10	0.01	0.05
Min-max	0.44–0.77	0.56–0.73	0.51–0.60	0.39–0.65	0.32–0.62	0.40–0.63	0.01–0.10	0.03–0.24	0.005–0.05	0.03–0.24

**Figure 7:**
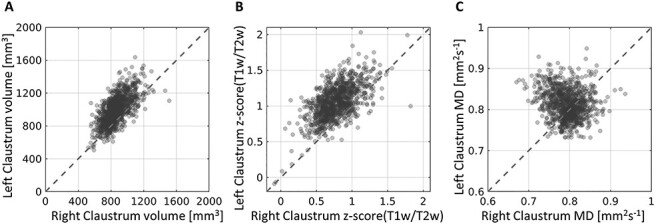
Claustrum characteristics. The left claustrum’s properties are plotted as a function of the right claustrum for all subjects. The volume (*A*) and the z-scored values of the T1w/T2w ratio (*B*) show high agreement between the left and right claustra, with the left claustrum showing higher values in both. The MD values (*C*) do not show a consistent difference between the left and right claustra across subjects. The mean values can be seen in Table 2.

To characterize the anatomical properties of the claustrum, we used our automatic segmentation to calculate in vivo estimates of tissue macro- and microstructure for each subject. We estimated the claustrum volume, the mean of the *z*-scored T1w/T2w values (which have been associated with myelin content), and the mean MD value (which is related to the tissue membrane content). The values of the left and right claustra for all subjects are plotted in Figure 7 for each measurement, and summarized in Table 2. We found that these properties of the claustrum span a relatively narrow range of values. The values of the left and right claustra are highly correlated in both volume and T1w/T2w measurements (but less so in MD), with higher values on the left. One possible explanation for this is the more diffuse structure of the left claustrum (see Discussion) would lead to more partial volume effects in the final segmentation, and hence to a greater volume (mean 995 and 863 mm^3^ for the left and right claustra, respectively). We did not find such volume lateralization in the manual segmentations of 10 subjects, as evident by the average volume across the 2 observers (mean 641 and 649 mm^3^ for the left and right claustra, respectively).

**Table 2:** Structural measurements of the automatically segmented claustrum. The mean and standard deviation of structural values measured for the left and right claustra across subjects. Key: a.u.: arbitrary units; L: left; R: right; std: standard deviation

	Volume [mm^3^]		T1w/T2w [a.u.]		MD [×10^−3^ mm^2^/s]	
	L	R	L	R	L	R
Mean	995	863	1.05	0.77	0.81	0.79
std	170	133	0.23	0.23	0.04	0.04

To test whether partial volume effects can account for the observed left–right difference, we repeated the measurements in the neighboring and much larger putamen, where partial volume effects are expected to have a much smaller effect on the measured values. Indeed, we found similar results in the putamen (Supplementary Fig. 1 and Supplementary Table 1), suggesting the partial volume effects do not fully account for the left–right difference we observed in the claustrum. The greater T1w/T2w values in the left hemisphere could also reflect a global residual bias in the contrast image. We note that for the putamen, we found slightly greater volume in the right hemisphere, in accordance with previous accounts (Abedelahi et al. 2013).

**Figure 8:**
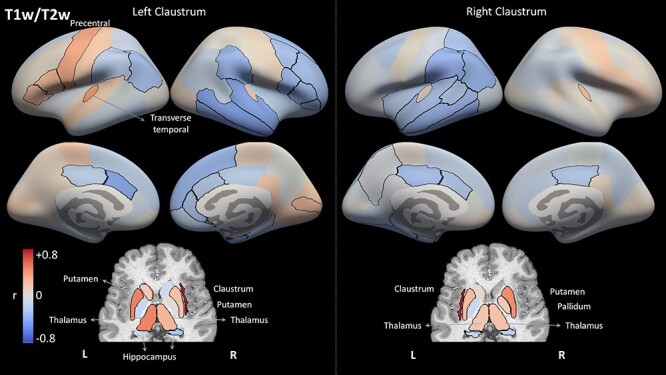
Structural correlation results for region volume. Each region is color-coded according to its correlation with the left or right claustrum in terms of volume. Regions with significant correlation (*p* <0.01, corrected for multiple comparisons of 2 hemispheres × 74 regions × 3 measures) are outlined in black. Note the overall symmetry between hemispheres, as well as between the results for the 2 claustra. Cortical regions are presented on FreeSurfer's inflated average cortical surface (top), whereas the subcortical regions are presented in an axial view (bottom). Key: L: left; R: right; r: Pearson's correlation coefficient.

**Figure 9:**
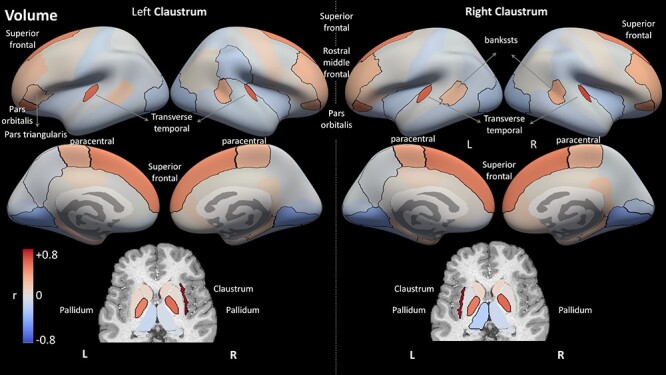
Structural correlation results for region T1w/T2w values. Each region is color-coded according to its correlation with the left or right claustrum in terms of standardized T1w/T2w values, similar to Figure 8. Compared with the results for volume (Fig. 8), the covariance network is less symmetrical, and consists of more negative correlations.

**Figure 10:**
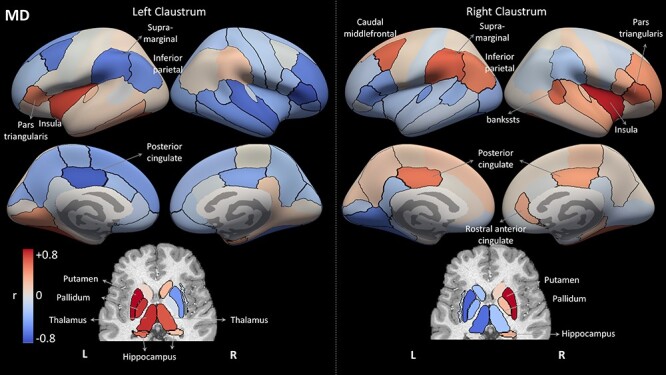
Structural correlation results for region MD values. Each region is color-coded according to its correlation with the left or right claustrum in terms of MD values, similar to Figure 8. Compared with the volume analysis (Fig. 8), the MD network is less symmetrical, and the MD values of bilateral claustra are not correlated with each other across subjects.

Finally, we used the 3 tissue measures to estimate the structural covariance of the claustrum with other brain regions across subjects. Figure 8 shows all brain regions colored according to their correlation with each claustrum in terms of volume. Figures 9 and 10 present results using the *z*-scored T1w/T2w values and the MD values, respectively. The different measures present distinct covariance networks, and each network is composed of widespread regions that include both subcortical and cortical regions. The correlation values and corresponding *P* values are presented in Supplementary Tables 2–4.

This volume covariance analysis presents the most symmetric pattern of correlations: The regions that covary with the left claustrum are mostly the same regions that covary with the right claustrum. Furthermore, the claustrum volume mostly covaries with the same regions in the left and right hemispheres. The prominent regions in the volume-based covariance network include the contralateral claustrum, the frontal regions of pars orbitalis and superior frontal cortex, the transverse temporal region, and the subcortical pallidum. For MD, the results for the left and right claustra are almost mirror images of each other, with regions positively correlated with the left claustrum showing negative correlation with the right claustrum (see Discussion for possible explanations of this result). For the T1w/T2w analysis, again we find similar results for the left and right claustra. The most strongly correlated regions were subcortical regions, predominantly the ipsilateral putamen and thalamus.

## Discussion

In this study, we developed an automatic segmentation of the left and right claustra on in vivo MRI data from the HCP. We validated the automatic segmentation using manual segmentation in 10 subjects and found good agreement. To demonstrate the utility of the automatic segmentation in studying the human claustrum, we calculated the structural covariance between the claustra and other gray-matter brain regions, using 3 measurements of tissue properties. The code is available at https://github.com/MezerLab/ClaustrumSegmentation and the resulting segmentations are available at https://doi.org/10.5281/zenodo.3960552.

Automatic segmentation of the claustrum is a prerequisite for large-scale studies of this intriguing structure. However, such automatic segmentation has proven to be a nontrivial task, as evidenced by the absence of the claustrum from commonly used tools for brain segmentation, such as FreeSurfer and FSL. Indeed, we could find only 1 published tool that automatically segments the claustrum, namely BrainSuite. Although BrainSuite’s segmentation is based on advanced surface-based volume registrations (Joshi et al. 2007), its segmentation of the claustrum remains unsatisfactory (Table 2). It should be noted that BrainSuite is an open-source tool with multiple functionalities and that segmentation of the claustrum is not its main focus.

To segment the dorsal claustrum successfully, we took a heuristic approach, based on gross anatomical landmarks and rooted in post mortem studies of the human claustrum. Importantly, we capitalize on the well-established segmentation of the putamen by FSL’s FIRST (Jenkinson and Smith 2001; Jenkinson et al. 2002). We used the putamen segmentation to mark the initial voxels from which the dorsal claustrum is later segmented. An alternative would have been to start at the lateral border, using a segmentation of the insula. However, the insular segmentation is relatively prone to overestimation errors and occasionally includes the neighboring white matter, or even parts of the claustrum (McCarthy et al. 2015). The ventral-dorsal division of the human claustrum has been identified in post mortem studies. These studies have shown that although the dorsal claustrum is a continuous structure, the ventral claustrum is a smaller structure composed of scattered islands of gray matter, in between fibers of the white matter (Druga 2014).

The automatic segmentations resulted in a dorsal claustrum whose mean volume is 863 mm^3^ for the right hemisphere and 995 mm^3^ for left. These values are greater than the ones we found in the manual segmentations of 10 subjects (649 and 641 mm^3^ for the right and left claustra, respectively), indicating that our automatic segmentation is permissive compared with the more conservative manual segmentations. The volumes we found for the automatic segmentation generally agree with those reported in another MRI study who used manual segmentation of the claustrum in 10 subjects, finding values of 813.6 mm^3^ (range 744–864) for the right claustrum and 804.0 mm^3^ (range 752–912) for the left claustrum (Milardi et al. 2015). Thus, although Milardi and colleagues found a smaller left claustrum, we found the opposite. One possible interpretation for this discrepancy is that the left claustrum is indeed smaller, leading to greater partial volume effects with the neighboring white matter, which in turn leads to an overestimation of the left claustrum’s volume in the proposed segmentation.

We compared the proposed segmentation to manual segmentations by 2 observers and found mean Dice coefficients of approximately 0.6. These values are much higher than those we found for the published method by BrainSuite. Dice coefficients of 0.51–0.58 are considered moderate for neuroimaging segmentations of subcortical regions, but this can be attributed to the claustrum’s small dimensions and its unusual shape. Dice coefficient usually decreases with structure size (Patenaude et al. 2011; Worth and Tourville 2015). In addition, many subcortical nuclei (such as the putamen and thalamus) have a roughly globular shape, such that different segmentation methods often agree on the innermost voxels at least. This is in contrast to the claustrum, whose thin curtain-like shape leads to a high surface-to-volume ratio, with a large fraction of voxels at the border, precisely where different segmentations may disagree.

Using correlations between structural measures in cortical and various subcortical regions, we identified several regions that covary with the claustrum across subjects. It has been postulated that structural covariance between regions could indicate that they are a part of the same functional network (Mechelli et al. 2005; Alexander-Bloch, Giedd, et al. 2013). Covariance analysis based on different measures need not yield similar networks (Alexander-Bloch, Giedd, et al. 2013). Indeed, our results did not reveal 1 consistent network that shows structural covariance with the claustrum across measures: Instead, each of the 3 measurements (volume, T1w/T2w, and MD) revealed a different set of regions that include contributions from different lobes, which are often implicated in various functional domains (as we discuss in detail below). Such analyses of structural covariance are complementary to other connectivity estimates based on dMRI tractography or rs-fMRI. Indeed, previous studies that described claustrum networks using fMRI or dMRI found extensive and dispersed connectivity patterns between the claustrum and other brain regions (Milardi et al. 2015; Torgerson et al. 2015; Krimmel et al. 2019). It should be noted that rs-fMRI and dMRI studies typically acquire data with lower spatial resolution (usually 2^3^–3^3^ mm^3^). This makes it difficult to disentangle the connectivity of the claustrum from that of its surroundings and requires specialized analysis techniques as recently proposed by Krimmel et al. (2019).

We found that the frontal and temporal cortices form a volume-based structural covariance network with the claustrum. As we describe in detail below, these results agree with previous findings in humans and in other species. In humans, these regions have been described as a part of the same resting state functional connectivity network (Krimmel et al. 2019). The connectivity between the human claustrum and frontal and orbital cortices has also been demonstrated in dMRI studies (Fernández-Miranda et al. 2008; Torgerson et al. 2015).

Homologous areas have been functionally and anatomically associated with the claustrum in other species, though cross-species homologies are not always obvious. Although the claustrum itself has been identified in many species, there is cross-species variability in its topographical arrangement and its defined anatomical boundary, including qualitative differences in regards to the ventral-dorsal separation (Kowiański et al. 1999; Baizer 2014). Therefore, below we refer to interesting correspondence between our results and animal studies without distinction between ventral and dorsal claustrum. In capuchins, tracing studies showed afferent connections to the orbitofrontal cortex from the claustrum (Reser et al. 2014). Bilateral connectivity between claustrum and orbitofrontal cortex is prominent in tracing studies in rodents as well (Atlan et al. 2017; Wang et al. 2017). Another approach for studying brain connectivity is using optogenetics, where optogenetic activation of the claustrum in mice was found to suppress activity in the frontal cortex (Jackson et al. 2018; White and Mathur 2018). In addition to frontal regions, we found connections between the claustrum and the transverse temporal cortex, which is part of the primary auditory cortex. Temporo-claustral connections have also been demonstrated in rodents tracing studies (Sadowski et al. 1997; Zingg et al. 2014; Atlan et al. 2017), as well as in cats (Olson and Graybiel 1980) and nonhuman primates (Remedios et al. 2010), where claustrum activity was recorded in response to auditory stimuli. Furthermore, optogenetic stimulation of the claustrum in mice can suppress evoked activity in the auditory cortex (Atlan et al. 2018). The parahippocampal region includes the entorhinal cortex, which is robustly interconnected with the rodent claustrum (Kerr et al. 2007; Zingg et al. 2014; Atlan et al. 2017; Wang et al. 2017). Finally, we found that the pallidum volume covaries with that of the claustrum. Although we could not find evidence for such connections in the literature, there is evidence suggesting connections between the claustrum and subcortical regions such as the putamen (Borra et al. 2020). In total, these results of volume-wise structural covariation in the current study recapitulate anatomical and functional observations of claustral connectivity across different species and different methodologies.

In this study, we further studied the claustrum’s structural covariance using 2 microstructural tissue measurements, namely T1w/T2w and MD. In contrast to the volume-based network, the microstructural-based networks were less symmetric and contained more regions with negative correlations across subjects. Although studying structural covariance with microstructural measurements has the potential to reveal network that are not found by volume covariance, these results should be interpreted with caution. T1w/T2w is a semiquantitative measure, potentially with subject-specific biases. Here, we addressed this issue by standardizing the regional measurements using *z*-score (akin to Ma and Zhang (2017)). Other approaches were proposed to account for such biases, like using partial correlation (Melie-Garcia et al. 2018) or adding confounding variables in a linear regression framework (Mechelli et al. 2005). Alternatively, 1 could employ quantitative MRI acquisitions, which include quantitative relaxometry such as T1 and T2, to shed more light on the claustrum’s microstructure-based networks. As highlighted recently, future studies could also investigate the reproducibility of the claustrum’s covariance networks under different methodological choices (Carmon et al. 2020).

Although MD is a quantitative measurement, the scan resolution of the diffusion weighted MRI data was lower than the anatomical scans (1.25 mm^3^ compared with 0.7 mm^3^). Furthermore, recent work suggests that gradient nonlinearities in the dMRI data of the HCP dataset could lead to biases of up to 10% in the estimated values (Mesri et al. 2020). This work showed that the bias is largest in the cerebellum and in frontal regions, and therefore should not affect the values in the claustrum itself too much. However, this nonlinearity could potentially contribute to our finding of a decreased left–right agreement in MD values compared with the other measures. As such, it might affect the results of our MD-based covariance analysis and explain the mirror-like results between the left and right claustra. Future work using the HCP dataset could employ a gradient nonlinearity correction to overcome this limitation (Bammer et al. 2003).

This work focuses on segmenting the dorsal claustrum, as did other works investigating the human claustrum in vivo (Fernández-Miranda et al. 2008; Torgerson et al. 2015; Krimmel et al. 2019). The fragmented nature of the ventral claustrum makes it challenging to segment reliably, even using the cutting-edge, high-resolution of the HCP dataset. Even with a higher spatial resolution of 0.5^3^ mm^3^, the ventral claustrum could be detected and segmented manually only in some subjects but not in others (Davis 2008).

Manual segmentation can be extremely time-consuming, especially for large datasets. In this study, we developed an algorithm to automatically segment the dorsal claustrum and successfully ran it on 1068 subjects. Our algorithm is designed for high-resolution data, which is becoming more prevalent in the scientific community. For example, the proposed algorithm could be used when the HCP’s development and aging datasets are fully released. We hope that the proposed claustrum segmentation algorithm, as well as the segmentations we made publicly available, will advance the study of this intriguing structure and help shed light on its function in humans.

## Supplementary Material

Claustrum_segmentation_supplementary_tgaa062

Claustrum_segmentation_3D_tgaa062

## Notes

We thank Yuval Benjamini for his helpful comments on the structural covariance analysis.

## References

[ref1] Abedelahi A, Hasanzadeh H, Hadizadeh H, Joghataie MT. 2013. Morphometric and volumetric study of caudate and putamen nuclei in normal individuals by MRI: effect of normal aging, gender and hemispheric differences. Polish J Radiol. 78:7–14. 10.12659/PJR.889364 PMC3789937 24115954

[ref2] Alexander-Bloch A, Giedd JN, Bullmore E. 2013. Imaging structural co-variance between human brain regions. Nat Rev Neurosci. 14:322–336. 23531697 10.1038/nrn3465 PMC4043276

[ref3] Alexander-Bloch A, Raznahan A, Bullmore E, Giedd J. 2013. The convergence of maturational change and structural covariance in human cortical networks. J Neurosci. 33:2889–2899. 23407947 10.1523/JNEUROSCI.3554-12.2013 PMC3711653

[ref4] Andersson JLR, Skare S, Ashburner J. 2003. How to correct susceptibility distortions in spin-echo echo-planar images: application to diffusion tensor imaging. NeuroImage. 20:870–888. 14568458 10.1016/S1053-8119(03)00336-7

[ref5] Andersson JLR, Sotiropoulos SN. 2016. An integrated approach to correction for off-resonance effects and subject movement in diffusion MR imaging. NeuroImage. 125:1063–1078. 26481672 10.1016/j.neuroimage.2015.10.019 PMC4692656

[ref6] Andrews TJ, Halpern SD, Purves D. 1997. Correlated size variations in human visual cortex, lateral geniculate nucleus, and optic tract. J Neurosci. 17:2859–2868. 9092607 10.1523/JNEUROSCI.17-08-02859.1997 PMC6573115

[ref7] Arshad M, Stanley JA, Raz N. 2017. Test-retest reliability and concurrent validity of in vivo myelin content indices: myelin water fraction and calibrated T _1_ w/T _2_ w image ratio. Hum Brain Mapp. 38:1780–1790. 28009069 10.1002/hbm.23481 PMC5342928

[ref8] Atlan G, Terem A, Peretz-Rivlin N, Groysman M, Citri A. 2017. Mapping synaptic cortico-claustral connectivity in the mouse. J Comp Neurol. 525:1381–1402. 26973027 10.1002/cne.23997

[ref9] Atlan G, Terem A, Peretz-Rivlin N, Sehrawat K, Gonzales BJ, Pozner G, Tasaka G, Goll Y, Refaeli R, Zviran O, Lim BK, et al. 2018. The claustrum supports resilience to distraction. Curr Biol. 28:2752–2762.e7. 30122531 10.1016/j.cub.2018.06.068 PMC6485402

[ref10] Baizer JS . 2014. The neurochemical organization of the claustrum. Smythies JR, Edelstein LR, Ramachandran VS, editors. The claustrum: structural, functional, and clinical neuroscience. San Diego (CA): Academic Press. p. 85–118.

[ref11] Bammer R, Markl M, Barnett A, Acar B, Alley MT, Pelc NJ, Glover GH, Moseley ME. 2003. Analysis and generalized correction of the effect of spatial gradient field distortions in diffusion-weighted imaging. Magn Reson Med. 50:560–569. 12939764 10.1002/mrm.10545

[ref12] Borra E, Luppino G, Gerbella M, Rozzi S, Rockland KS. 2020. Projections to the putamen from neurons located in the white matter and the claustrum in the macaque. J Comp Neurol. 528:453–467. 31483857 10.1002/cne.24768 PMC6901742

[ref13] Carmon J, Heege J, Necus JH, Owen TW, Pipa G, Kaiser M, Taylor PN, Wang Y. 2020. Reliability and comparability of human brain structural covariance networks. NeuroImage. 220:117104. 32621973 10.1016/j.neuroimage.2020.117104

[ref14] Crick FC, Koch C. 2005. What is the function of the claustrum? Philos Trans R Soc B Biol Sci. 360:1271–1279. 10.1098/rstb.2005.1661 PMC1569501 16147522

[ref15] Davis W. 2008. The claustrum in autism and typically developing male children: a quantitative MRI study. Brigham Young University.

[ref16] Desikan RS, Ségonne F, Fischl B, Quinn BT, Dickerson BC, Blacker D, Buckner RL, Dale AM, Maguire RP, Hyman BT, et al. 2006. An automated labeling system for subdividing the human cerebral cortex on MRI scans into gyral based regions of interest. NeuroImage. 31:968–980. 16530430 10.1016/j.neuroimage.2006.01.021

[ref17] Dice LR . 1945. Measures of the amount of ecologic association between species. Ecology. 26:297–302.

[ref18] Dickstein SG, Bannon K, Xavier Castellanos F, Milham MP. 2006. The neural correlates of attention deficit hyperactivity disorder: an ALE meta-analysis. J Child Psychol Psychiatry Allied Discip. 47:1051–1062. 10.1111/j.1469-7610.2006.01671.x 17073984

[ref19] Druga R. 2014. The structure and connections of the claustrum. In: Smythies JR, Edelstein LR, Ramachandran VS, editors. The Claustrum: Structural, Functional, and Clinical Neuroscience. San Diego (CA): Academic Press. p. 29–84.

[ref20] DuPre E, Spreng RN. 2017. Structural covariance networks across the life span, from 6 to 94 years of age. Netw Neurosci. 1:302–323. 29855624 10.1162/NETN_a_00016 PMC5874135

[ref21] Edelstein LR, Denaro FJ. 2004. The claustrum: a historical review of its anatomy, physiology, cytochemistry and functional significance. Cell Mol Biol (Noisy-le-grand). 50:675–702. 15643691

[ref22] Ersche KD, Jones PS, Williams GB, Turton AJ, Robbins TW, Bullmore ET. 2012. Abnormal brain structure implicated in stimulant drug addiction. Science. 335:601–604. 22301321 10.1126/science.1214463

[ref23] Fassbender C, Schweitzer JB, Cortes CR, Tagamets MA, Windsor TA, Reeves GM, Gullapalli R. 2011. Working memory in attention deficit/hyperactivity disorder is characterized by a lack of specialization of brain function. PLoS One. 6:e27240. 10.1371/journal.pone.0027240 PMC3213127 22102882

[ref24] Fernández-Miranda JC, Rhoton AL, Kakizawa Y, Choi C, Álvarez-Linera J. 2008. The claustrum and its projection system in the human brain: a microsurgical and tractographic anatomical study - laboratory investigation. J Neurosurg. 108:764–774. 18377257 10.3171/JNS/2008/108/4/0764

[ref25] Glasser MF, Sotiropoulos SN, Wilson JA, Coalson TS, Fischl B, Andersson JL, Xu J, Jbabdi S, Webster M, Polimeni JR, et al. 2013. The minimal preprocessing pipelines for the human connectome project. NeuroImage. 80:105–124. 23668970 10.1016/j.neuroimage.2013.04.127 PMC3720813

[ref26] Glasser MF, Van Essen DC. 2011. Mapping human cortical areas in vivo based on myelin content as revealed by T1- and T2-weighted MRI. J Neurosci. 31:11597–11616. 21832190 10.1523/JNEUROSCI.2180-11.2011 PMC3167149

[ref27] Goll Y, Atlan G, Citri A. 2015. Attention: the claustrum. Trends Neurosci. 38:486–495. 26116988 10.1016/j.tins.2015.05.006

[ref28] Gong G, He Y, Chen ZJ, Evans AC. 2012. Convergence and divergence of thickness correlations with diffusion connections across the human cerebral cortex. NeuroImage. 59:1239–1248. 21884805 10.1016/j.neuroimage.2011.08.017

[ref29] Hall MG, Alexander DC. 2009. Convergence and parameter choice for Monte-Carlo simulations of diffusion MRI. IEEE Trans Med Imaging. 28:1354–1364. 19273001 10.1109/TMI.2009.2015756

[ref30] Heimer L, de Olmos JS, Alheid GF, Pearson J, Sakamoto N, Shinoda K, Marksteiner J, Switzer RC. 1999. Chapter II The human basal forebrain. Part II. In: Handbook of chemical neuroanatomy. Amsterdam, The Netherlands: Elsevier. p. 57–226.

[ref31] Hinova-Palova DV, Edelstein L, Landzhov B, Minkov M, Malinova L, Hristov S, Denaro FJ, Alexandrov A, Kiriakova T, Brainova I, et al. 2014. Topographical distribution and morphology of NADPH-diaphorase-stained neurons in the human claustrum. Front Syst Neurosci. 8:96. 10.3389/fnsys.2014.00096 PMC4034338 24904317

[ref32] Jackson J, Karnani MM, Zemelman BV, Burdakov D, Lee AK. 2018. Inhibitory control of prefrontal cortex by the claustrum. Neuron. 99:1029–1039.e4. 30122374 10.1016/j.neuron.2018.07.031 PMC6168643

[ref33] Jenkinson M, Bannister P, Brady M, Smith S. 2002. Improved optimization for the robust and accurate linear registration and motion correction of brain images. NeuroImage. 17:825–841. 12377157 10.1016/s1053-8119(02)91132-8

[ref34] Jenkinson M, Smith S. 2001. A global optimisation method for robust affine registration of brain images. Med Image Anal. 5:143–156. 11516708 10.1016/s1361-8415(01)00036-6

[ref35] Johnson JI, Fenske BA, Jaswa AS, Morris JA. 2014. Exploitation of puddles for breakthroughs in claustrum research. Front Syst Neurosci. 8:78. 10.3389/fnsys.2014.00078 PMC4030192 24860441

[ref36] Joshi AA, Shattuck DW, Thompson PM, Leahy RM. 2007. Surface-constrained volumetric brain registration using harmonic mappings. IEEE Trans Med Imaging. 26:1657–1669. 18092736 10.1109/tmi.2007.901432 PMC4516139

[ref37] Kalaitzakis ME . 2014. Parkinson’s disease and the claustrum. In: Smythies JR, Edelstein LR, Ramachandran VS, editors. The claustrum: structural, functional, and clinical neuroscience. San Diego (CA): Academic Press. p. 277–297.

[ref38] Kelly C, Toro R, Di Martino A, Cox CL, Bellec P, Castellanos FX, Milham MP. 2012. A convergent functional architecture of the insula emerges across imaging modalities. NeuroImage. 61:1129–1142. 22440648 10.1016/j.neuroimage.2012.03.021 PMC3376229

[ref39] Kerr KM, Agster KL, Furtak SC, Burwell RD. 2007. Functional neuroanatomy of the parahippocampal region: the lateral and medial entorhinal areas. Hippocampus. 17:697–708. 17607757 10.1002/hipo.20315

[ref40] Koubeissi MZ, Bartolomei F, Beltagy A, Picard F. 2014. Electrical stimulation of a small brain area reversibly disrupts consciousness. Epilepsy Behavior. 37:32–35. 24967698 10.1016/j.yebeh.2014.05.027

[ref41] Kowiański P, Dziewiatkowski J, Kowiańska J, Moryś J. 1999. Comparative anatomy of the claustrum in selected species: a morphometric analysis. Brain Behav Evol. 53:44–54. 9858804 10.1159/000006581

[ref42] Krimmel SR, White MG, Panicker MH, Barrett FS, Mathur BN, Seminowicz DA. 2019. Resting state functional connectivity and cognitive task-related activation of the human claustrum. NeuroImage. 196:59–67. 30954711 10.1016/j.neuroimage.2019.03.075 PMC6629463

[ref43] Le Bihan D, Mangin JF, Poupon C, Clark CA, Pappata S, Molko N, Chabriat H. 2001. Diffusion tensor imaging: concepts and applications. J Magn Reson Imaging. 13:534–546. 11276097 10.1002/jmri.1076

[ref44] Ma Z, Zhang N. 2017. Cross-population myelination covariance of human cerebral cortex. Hum Brain Mapp. 38:4730–4743. 28631354 10.1002/hbm.23698 PMC5547576

[ref45] Mathur BN . 2014. The claustrum in review. Front Syst Neurosci. 8:48. 24772070 10.3389/fnsys.2014.00048 PMC3983483

[ref46] McCarthy CS, Ramprashad A, Thompson C, Botti J, Coman IL, Kates WR. 2015. A comparison of FreeSurfer-generated data with and without manual intervention. Front Neurosci. 9:379. 10.3389/fnins.2015.00379 PMC4612506 26539075

[ref47] Mechelli A, Friston KJ, Frackowiak RS, Price CJ. 2005. Structural covariance in the human cortex. J Neurosci. 25:8303–8310. 16148238 10.1523/JNEUROSCI.0357-05.2005 PMC6725541

[ref48] Melie-Garcia L, Slater D, Ruef A, Sanabria-Diaz G, Preisig M, Kherif F, Draganski B, Lutti A. 2018. Networks of myelin covariance. Hum Brain Mapp. 39:1532–1554. 29271053 10.1002/hbm.23929 PMC5873432

[ref49] Mesri HY, David S, Viergever MA, Leemans A. 2020. The adverse effect of gradient nonlinearities on diffusion MRI: from voxels to group studies. NeuroImage. 205:116127. 31476431 10.1016/j.neuroimage.2019.116127

[ref50] Milardi D, Bramanti P, Milazzo C, Finocchio G, Arrigo A, Santoro G, Trimarchi F, Quartarone A, Anastasi G, Gaeta M. 2015. Cortical and subcortical connections of the human claustrum revealed in vivo by constrained spherical deconvolution tractography. Cerebral Cortex. 25:406–414. 24014669 10.1093/cercor/bht231

[ref51] Morel A, Gallay MN, Baechler A, Wyss M, Gallay DS. 2013. The human insula: architectonic organization and postmortem MRI registration. Neuroscience. 236:117–135. 23340245 10.1016/j.neuroscience.2012.12.076

[ref52] Morys J, Bobinski M, Wegiel J, Wisniewski HM, Narkiewicz O. 1996. Alzheimer’s disease severely affects areas of the claustrum connected with the entorhinal cortex. J Brain Res. 37:173–180. 8776503

[ref53] Olson CR, Graybiel AM. 1980. Sensory maps in the claustrum of the cat. Nature. 288:479–481. 7442793 10.1038/288479a0

[ref54] Patenaude B, Smith SM, Kennedy DN, Jenkinson M. 2011. A Bayesian model of shape and appearance for subcortical brain segmentation. NeuroImage. 56:907–922. 21352927 10.1016/j.neuroimage.2011.02.046 PMC3417233

[ref55] Rae ASL . 1954. The form and structure of the human claustrum. J Comp Neurol. 100:15–39. 13130707 10.1002/cne.901000103

[ref56] Remedios R, Logothetis NK, Kayser C. 2010. Unimodal responses prevail within the multisensory claustrum. J Neurosci. 30:12902–12907. 20881109 10.1523/JNEUROSCI.2937-10.2010 PMC6633510

[ref57] Reser DH, Richardson KE, Montibeller MO, Zhao S, Chan JMH, Soares JGM, Chaplin TA, Gattass R, Rosa MGP. 2014. Claustrum projections to prefrontal cortex in the capuchin monkey (Cebus apella). Front Syst Neurosci. 8:23. 10.3389/fnsys.2014.00123 PMC4079979 25071475

[ref58] Romero-Garcia R, Whitaker KJ, Váša F, Seidlitz J, Shinn M, Fonagy P, Dolan RJ, Jones PB, Goodyer IM, Bullmore ET, et al. 2018. Structural covariance networks are coupled to expression of genes enriched in supragranular layers of the human cortex. NeuroImage 171:256–267. 29274746 10.1016/j.neuroimage.2017.12.060 PMC5883331

[ref59] Sadowski M, Moryś J, Jakubowska-Sadowska K, Narkiewicz O. 1997. Rat’s claustrum shows 2 main cortico-related zones. Brain Research 756:147–152. 9187325 10.1016/s0006-8993(97)00135-2

[ref60] Sakamoto N, Pearson J, Shinoda K, Alheid GF, Olmos JSDE. 1999. The human basal forebrain. Part I. an overview. Science. 15:1–55.

[ref61] Smith JB, Alloway KD. 2014. Interhemispheric claustral circuits coordinate sensory and motor cortical areas that regulate exploratory behaviors. Front Syst Neurosci. 8:93. 24904315 10.3389/fnsys.2014.00093 PMC4032913

[ref62] Smith JB, Liang Z, Watson GDR, Alloway KD, Zhang N. 2017. Interhemispheric resting-state functional connectivity of the claustrum in the awake and anesthetized states. Brain Struct Funct. 222:2041–2058. 27714529 10.1007/s00429-016-1323-9 PMC5382132

[ref63] Smith RE, Tournier J, Calamante F, Connelly A. 2012. Anatomically-constrained tractography: improved diffusion MRI streamlines tractography through effective use of anatomical information. NeuroImage. 62:1924–1938. 22705374 10.1016/j.neuroimage.2012.06.005

[ref64] Snider SB, Hsu J, Darby RR, Cooke D, Fischer D, Cohen AL, Grafman JH, Fox MD. 2020. Cortical lesions causing loss of consciousness are anticorrelated with the dorsal brainstem. Hum Brain Mapp. 41:1520–1531. 31904898 10.1002/hbm.24892 PMC7268053

[ref65] Torgerson CM, Irimia A, Goh SYM, Van Horn JD. 2015. The DTI connectivity of the human claustrum. Hum Brain Mapp. 36:827–838. 25339630 10.1002/hbm.22667 PMC4324054

[ref66] Tournier J-D, Smith R, Raffelt D, Tabbara R, Dhollander T, Pietsch M, Christiaens D, Jeurissen B, Yeh C-H, Connelly A. 2019. MRtrix3: a fast, flexible and open software framework for medical image processing and visualisation. NeuroImage. 202:116137. 31473352 10.1016/j.neuroimage.2019.116137

[ref67] Uddin MN, Figley TD, Marrie RA, Figley CR. 2018. Can T1w/T2w ratio be used as a myelin-specific measure in subcortical structures? Comparisons between FSE-based T1w/T2w ratios, GRASE-based T1w/T2w ratios and multi-echo GRASE-based myelin water fractions. NMR Biomed. 31:e3868. 10.1002/nbm.3868 29315894

[ref68] Van Essen DC, Smith SM, Barch DM, Behrens TEJ, Yacoub E, Ugurbil K. 2013. The WU-Minn human connectome project: an overview. NeuroImage. 80:62–79. 23684880 10.1016/j.neuroimage.2013.05.041 PMC3724347

[ref69] Wang Q, Ng L, Harris JA, Feng D, Li Y, Royall JJ, Oh SW, Bernard A, Sunkin SM, Koch C, et al. 2017. Organization of the connections between claustrum and cortex in the mouse. J Comp Neurol. 525:1317–1346. 27223051 10.1002/cne.24047 PMC5324679

[ref70] Wang X, Wu Q, Egan L, Gu X, Liu P, Gu H, Yang Y, Luo J, Wu Y, Gao Z, et al. 2019. Anterior insular cortex plays a critical role in interoceptive attention. eLife 8:e42265. 10.7554/eLife.42265 PMC6488299 30985277

[ref71] Wei Y, Scholtens LH, Turk E, Van Den Heuvel MP. 2018. Multiscale examination of cytoarchitectonic similarity and human brain connectivity. Netw Neurosci. 3:124–137. 30793077 10.1162/netn_a_00057 PMC6372019

[ref72] White MG, Mathur BN. 2018. Frontal cortical control of posterior sensory and association cortices through the claustrum. Brain Struct Funct. 223:2999–3006. 29623428 10.1007/s00429-018-1661-x PMC5995986

[ref73] Worth A, Tourville J. 2015. Acceptable values of similarity coefficients in neuroanatomical labeling in MRI. Program No. 829.21, 2015 Meeting Planner. Chicago (IL): Society for Neuroscience. Online.

[ref74] Ye C, Albert M, Brown T, Bilgel M, Hsu J, Ma T, Caffo B, Miller MI, Mori S, Oishi K. 2019. Extended multimodal whole-brain anatomical covariance analysis: detection of disrupted correlation networks related to amyloid deposition. Heliyon. 5:e02074. 31372540 10.1016/j.heliyon.2019.e02074 PMC6656959

[ref75] Zingg B, Hintiryan H, Gou L, Song MY, Bay M, Bienkowski MS, Foster NN, Yamashita S, Bowman I, Toga AW, et al. 2014. Neural networks of the mouse neocortex. Cell. 156:1096–1111. 24581503 10.1016/j.cell.2014.02.023 PMC4169118

