# Supplementary material for: Automatic Segmentation of the Dorsal Claustrum in Humans Using in vivo High-Resolution MRI
Source: Cereb Cortex Commun. 2020 Sep 1;1(1):tgaa062. doi: 10.1093/texcom/tgaa062 (PMC8153060; doi:10.1093/texcom/tgaa062)
Supplement: Claustrum_segmentation_supplementary_tgaa062 [file claustrum_segmentation_supplementary_tgaa062.pdf]

Supplementary material for:

Automatic segmentation of the dorsal claustrum in humans using *in vivo* high-resolution MRI

Shai Berman[1]*, Roey Schurr[1]*†, Gal Atlan[1], Ami Citri[1,2,3], Aviv A. Mezer[1]

[1] Edmond and Lily Safra Center for Brain Sciences, Hebrew University of Jerusalem, Jerusalem, Israel

[2] Institute of Life Sciences, Edmond J. Safra Campus, Hebrew University of Jerusalem, Jerusalem, Israel

[3] Program in Child and Brain Development, Canadian Institute for Advanced Research, Toronto, ON M5G 1M1, Canada

* These authors contributed equally to this work

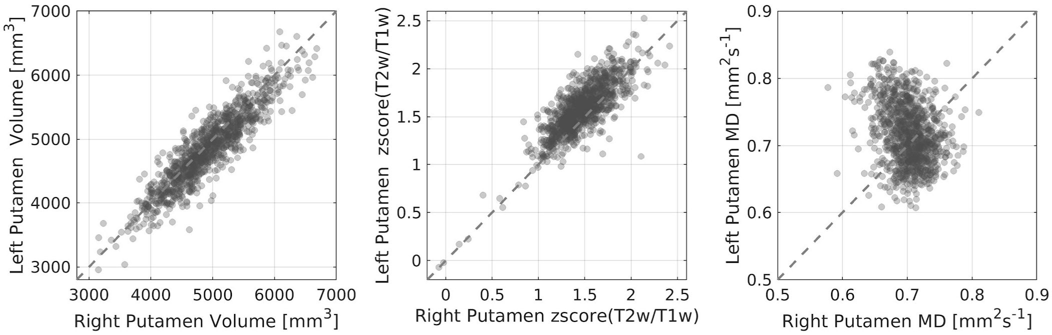

Supplementary Figure 1: Putamen characteristics. The properties of the left putamen are plotted as a function of the right putamen for all subjects. The volume (A) and the z-scored values of the T1w/T2w ratio (B) show high correlation between the left and right claustra with the left putamen showing a smaller volume but higher T1w/T2w values. The MD values (C) do not show a consistent difference between the left and right putamen. See Supplementary Table 1.

	Volume [mm^3]		T1w/T2w [a.u.]		MD [x10^{-3} mm^2/s]	
	**L**	**R**	**L**	**R**	**L**	**R**
**mean**	4839.4	4916.6	1.58	1.47	0.72	0.70
**std**	565.1	572.06	0.26	0.26	0.045	0.029

Supplementary Table 1: Structural measurements of the putamen. The mean and standard deviation of structural values measured for the left and right putamen across subjects. Key: a.u.: arbitrary units; L: left; R: right; std: standard deviation.

	**Left Claustrum**				**Right Claustrum**			
	ROI	r	p		ROI	r	p	
**1**	rh claustrum	0.80	3.05E-243	*	lh claustrum	0.80	3.05E-243	*
**2**	lh frontalpole	0.57	4.99E-92	*	lh frontalpole	0.63	2.65E-117	*
**3**	rh frontalpole	0.51	1.14E-70	*	rh frontalpole	0.56	4.20E-90	*
**4**	rh transversetemporal	0.44	7.79E-52	*	rh transversetemporal	0.51	8.06E-73	*
**5**	rh pallidum	0.43	9.24E-49	*	rh pallidum	0.44	3.32E-52	*
**6**	lh transversetemporal	0.40	1.22E-41	*	lh transversetemporal	0.42	9.74E-47	*
**7**	lh pallidum	0.39	1.55E-39	*	lh pallidum	0.40	2.03E-41	*
**8**	lh parsorbitalis	0.34	3.15E-30	*	lh parsorbitalis	0.35	1.10E-32	*
**9**	lh superiorfrontal	0.32	2.76E-27	*	rh superiorfrontal	0.34	6.60E-31	*
**10**	rh superiorfrontal	0.31	2.55E-25	*	lh superiorfrontal	0.34	8.83E-31	*
**11**	rh lingual	-0.29	5.81E-22	*	rh lingual	-0.31	7.84E-25	*
**12**	lh lingual	-0.27	5.41E-19	*	lh lingual	-0.28	2.36E-21	*
**13**	rh parsorbitalis	0.24	1.96E-15	*	rh parsorbitalis	0.28	1.99E-20	*
**14**	lh fusiform	-0.18	1.69E-09	*	lh paracentral	0.21	1.46E-12	*
**15**	lh paracentral	0.18	2.49E-09	*	rh bankssts	0.21	1.07E-11	*
**16**	rh bankssts	0.18	6.33E-09	*	rh rostralmiddlefrontal	0.19	2.34E-10	*
**17**	rh paracentral	0.17	5.06E-08	*	rh lateraloccipital	-0.19	8.03E-10	*
**18**	rh parahippocampal	0.17	5.63E-08	*	lh fusiform	-0.18	8.54E-09	*
**19**	rh lateraloccipital	-0.16	2.59E-07	*	rh paracentral	0.17	9.42E-09	*
**20**	rh rostralmiddlefrontal	0.15	6.76E-07	*	lh rostralmiddlefrontal	0.16	1.42E-07	*
**21**	lh precuneus	-0.15	8.63E-07	*	lh precuneus	-0.16	1.45E-07	*
**22**	rh supramarginal	-0.14	6.43E-06	*	lh thalamus proper	-0.15	3.72E-07	*
**23**	rh fusiform	-0.14	8.02E-06	*	rh insula	-0.15	4.04E-07	*
**24**	rh insula	-0.14	9.40E-06	*	lh lateraloccipital	-0.15	6.18E-07	*
**25**	lh parstriangularis	0.13	1.29E-05	*	rh parahippocampal	0.15	1.04E-06	*
**26**	lh lateraloccipital	-0.13	1.67E-05	*	lh bankssts	0.15	1.34E-06	*

**27**	lh parahippocampal	0.13	4.13E-05	lh entorhinal	0.14	5.82E-06	*
**28**	rh pericalcarine	-0.12	5.37E-05	rh fusiform	-0.14	6.99E-06	*
**29**	lh rostralmiddlefrontal	0.12	9.56E-05	rh pericalcarine	-0.13	1.79E-05	*
**30**	lh bankssts	0.11	2.78E-04	lh insula	-0.13	1.85E-05	*
**31**	lh lateralorbitofrontal	-0.11	4.17E-04	lh temporalpole	0.12	7.26E-05	
**32**	lh postcentral	-0.11	4.91E-04	lh parahippocampal	0.12	1.64E-04	
**33**	rh precuneus	-0.10	5.96E-04	rh entorhinal	0.11	2.20E-04	
**34**	rh superiorparietal	-0.10	0.001	lh lateralorbitofrontal	-0.11	2.28E-04	
**35**	lh middletemporal	-0.10	0.002	lh parstriangularis	0.11	2.97E-04	
**36**	lh insula	-0.09	0.002	lh postcentral	-0.11	3.74E-04	
**37**	lh pericalcarine	-0.09	0.002	lh pericalcarine	-0.10	6.76E-04	
**38**	lh thalamus proper	-0.09	0.003	rh middletemporal	-0.10	7.39E-04	
**39**	rh middletemporal	-0.09	0.003	rh isthmuscingulate	0.10	7.84E-04	
**40**	rh superiortemporal	-0.09	0.005	rh supramarginal	-0.10	8.74E-04	
**41**	rh lateralorbitofrontal	-0.08	0.009	lh middletemporal	-0.10	9.94E-04	
**42**	lh entorhinal	0.08	0.012	rh precuneus	-0.10	0.002	
**43**	rh inferiortemporal	-0.07	0.015	lh inferiorparietal	-0.09	0.002	
**44**	lh inferiortemporal	-0.07	0.024	rh lateralorbitofrontal	-0.09	0.002	
**45**	rh rostralanteriorcingulate	0.07	0.024	rh inferiortemporal	-0.09	0.002	
**46**	rh entorhinal	0.07	0.026	rh superiorparietal	-0.09	0.002	
**47**	lh caudate	0.07	0.026	lh inferiortemporal	-0.09	0.004	
**48**	rh isthmuscingulate	0.07	0.027	rh temporalpole	0.09	0.004	
**49**	rh caudate	0.07	0.028	rh thalamus proper	-0.08	0.011	
**50**	lh inferiorparietal	-0.07	0.029	rh medialorbitofrontal	0.07	0.027	
**51**	lh putamen	0.07	0.031	lh caudate	0.07	0.029	
**52**	lh medialorbitofrontal	-0.06	0.043	rh caudate	0.06	0.039	
**53**	rh parstriangularis	0.06	0.044	rh rostralanteriorcingulate	0.06	0.040	
**54**	rh postcentral	-0.06	0.052	lh superiorparietal	-0.05	0.083	
**55**	lh supramarginal	-0.06	0.062	rh superiortemporal	-0.05	0.084	
**56**	rh hippocampus	-0.06	0.068	lh supramarginal	-0.05	0.100	
**57**	rh precentral	0.05	0.076	rh caudalmiddlefrontal	-0.05	0.102	
**58**	lh cuneus	-0.05	0.108	lh posteriorcingulate	0.05	0.121	
**59**	lh superiortemporal	-0.04	0.157	lh cuneus	-0.05	0.124	
**60**	lh temporalpole	0.04	0.157	lh hippocampus	-0.04	0.155	
**61**	rh cuneus	-0.04	0.165	rh postcentral	-0.04	0.158	
**62**	rh caudalmiddlefrontal	-0.04	0.171	rh cuneus	-0.04	0.208	
**63**	lh posteriorcingulate	0.04	0.176	rh hippocampus	-0.04	0.210	
**64**	rh thalamus proper	-0.04	0.217	lh medialorbitofrontal	-0.04	0.215	
**65**	lh hippocampus	-0.03	0.266	rh parstriangularis	0.03	0.257	
**66**	lh caudalanteriorcingulate	-0.03	0.275	lh superiortemporal	-0.03	0.336	
**67**	rh putamen	0.03	0.312	lh caudalmiddlefrontal	-0.03	0.341	
**68**	lh superiorparietal	-0.03	0.334	rh putamen	0.02	0.571	
**69**	lh isthmuscingulate	-0.02	0.624	lh parsopercularis	-0.02	0.603	

**70**	rh medialorbitofrontal	0.01	0.638		lh precentral	-0.01	0.651	
**71**	lh rostralanteriorcingulate	-0.01	0.658		rh inferiorparietal	-0.01	0.661	
**72**	rh posteriorcingulate	-0.01	0.748		rh parsopercularis	-0.01	0.677	
**73**	rh parsopercularis	0.01	0.804		rh caudalanteriorcingulate	0.01	0.774	
**74**	lh caudalmiddlefrontal	0.01	0.827		lh isthmuscingulate	-0.01	0.788	
**75**	rh inferiorparietal	0.01	0.838		rh posteriorcingulate	-0.01	0.805	
**76**	lh parsopercularis	0.00	0.890		lh putamen	-0.01	0.853	
**77**	lh precentral	0.00	0.921		lh caudalanteriorcingulate	0.00	0.904	
**78**	rh caudalanteriorcingulate	0.00	0.946		rh precentral	0.00	0.928	
**79**	rh temporalpole	0.00	0.967		lh rostralanteriorcingulate	0.00	0.950	

* p<0.01 (Bonferroni corrected for multiple comparisons)

Supplementary Table 2. Structural covariance analysis for volume. Pearson correlation coefficients between the claustrum and other brain regions are presented alongside the uncorrected p-values, across N=1068 subjects. Regions names are listed using their according to their absolute correlation value in descending order. Statistical significance (p<0.01) is indicated by an asterisk, after Bonferroni correction for multiple comparisons (474 comparisons: 2 hemispheres x 79 regions x 3 measures). The names of the cortical regions are listed according to the FreeSurfer labels of the Desikan-Killiany atlas. lh: left hemisphere, rh:right hemisphere.

	**Left Claustrum**				**Right Claustrum**			
	ROI	r	p		ROI	r	p	
**1**	rh claustrum	0.60	0	*	lh claustrum	0.60	0	*
**2**	rh temporalpole	-0.38	5.65E-38	*	rh putamen	0.34	1.68E-30	*
**3**	lh putamen	0.38	1.31E-37	*	lh supramarginal	-0.26	1.48E-17	*
**4**	lh thalamus proper	0.36	6.46E-35	*	lh inferiorparietal	-0.25	1.00E-16	*
**5**	rh middletemporal	-0.32	3.05E-27	*	lh inferiortemporal	-0.25	1.04E-16	*
**6**	rh superiortemporal	-0.31	6.30E-25	*	lh middletemporal	-0.23	3.42E-14	*
**7**	lh caudalanteriorcingulate	-0.31	1.45E-24	*	lh fusiform	-0.22	1.26E-13	*
**8**	rh inferiortemporal	-0.28	4.44E-20	*	lh precuneus	-0.22	1.41E-13	*
**9**	rh entorhinal	-0.26	1.14E-17	*	lh posteriorcingulate	-0.21	5.27E-12	*
**10**	lh transversetemporal	0.26	1.77E-17	*	lh putamen	0.20	1.82E-11	*
**11**	rh supramarginal	-0.24	1.66E-15	*	lh temporalpole	-0.20	5.21E-11	*
**12**	rh parsopercularis	-0.24	3.44E-15	*	lh superiortemporal	-0.20	7.41E-11	*
**13**	rh superiorfrontal	-0.23	2.96E-14	*	lh superiorparietal	-0.19	1.80E-10	*
**14**	rh hippocampus	-0.22	1.53E-13	*	rh hippocampus	-0.17	1.30E-08	*
**15**	rh thalamus proper	0.22	3.42E-13	*	rh posteriorcingulate	-0.17	2.08E-08	*
**16**	lh precentral	0.22	4.10E-13	*	rh thalamus proper	0.17	2.94E-08	*
**17**	rh parsorbitalis	-0.22	1.01E-12	*	lh caudalanteriorcingulate	-0.17	4.53E-08	*
**18**	rh caudalmiddlefrontal	-0.21	3.14E-12	*	lh thalamus proper	0.16	1.27E-07	*

**19**	rh rostralmiddlefrontal	-0.21	5.16E-12	*	lh bankssts	-0.16	1.84E-07	*
**20**	rh parstriangularis	-0.20	1.40E-11	*	lh entorhinal	-0.15	3.80E-07	*
**21**	lh inferiorparietal	-0.20	1.60E-11	*	rh pallidum	0.15	8.13E-07	*
**22**	lh temporalpole	-0.20	2.62E-11	*	lh insula	-0.15	1.63E-06	*
**23**	rh rostralanteriorcingulate	-0.19	7.78E-10	*	lh parahippocampal	-0.14	3.11E-06	*
**24**	rh putamen	0.19	1.11E-09	*	rh transversetemporal	0.14	6.59E-06	*
**25**	lh parsopercularis	0.17	1.82E-08	*	lh hippocampus	-0.13	9.68E-06	*
**26**	lh parstriangularis	0.17	3.36E-08	*	lh caudalmiddlefrontal	-0.13	3.54E-05	
**27**	rh pericalcarine	0.16	1.39E-07	*	rh caudalanteriorcingulate	-0.12	1.19E-04	
**28**	rh caudalanteriorcingulate	-0.15	1.76E-06	*	rh temporalpole	-0.12	1.23E-04	
**29**	lh caudate	0.14	4.27E-06	*	rh precuneus	-0.11	2.34E-04	
**30**	lh posteriorcingulate	-0.14	5.11E-06	*	rh precentral	0.11	2.71E-04	
**31**	rh parahippocampal	-0.13	1.33E-05	*	rh rostralanteriorcingulate	-0.11	3.06E-04	
**32**	lh supramarginal	-0.13	1.73E-05	*	rh parsopercularis	0.11	3.14E-04	
**33**	rh posteriorcingulate	-0.13	2.41E-05		rh parstriangularis	0.11	4.83E-04	
**34**	rh insula	-0.13	2.55E-05		lh superiorfrontal	-0.11	5.21E-04	
**35**	lh postcentral	0.12	5.24E-05		rh supramarginal	-0.10	0.001	
**36**	lh precuneus	-0.12	5.30E-05		lh pallidum	-0.09	0.004	
**37**	lh paracentral	0.12	6.46E-05		lh lingual	-0.09	0.004	
**38**	lh inferiortemporal	-0.12	8.02E-05		lh parsopercularis	-0.08	0.006	
**39**	rh isthmuscingulate	0.12	8.39E-05		rh inferiorparietal	-0.08	0.008	
**40**	rh medialorbitofrontal	-0.12	1.06E-04		rh superiorfrontal	-0.08	0.010	
**41**	rh lateralorbitofrontal	-0.12	1.24E-04		rh parsorbitalis	0.08	0.013	
**42**	lh superiorfrontal	-0.12	1.54E-04		lh lateraloccipital	-0.07	0.015	
**43**	rh fusiform	-0.11	2.26E-04		lh caudate	0.07	0.016	
**44**	rh cuneus	0.11	3.95E-04		rh superiorparietal	-0.07	0.023	
**45**	rh bankssts	-0.11	5.78E-04		lh postcentral	-0.07	0.023	
**46**	lh hippocampus	-0.10	7.73E-04		rh lateralorbitofrontal	0.07	0.028	
**47**	rh lingual	0.10	7.83E-04		lh rostralmiddlefrontal	-0.07	0.028	
**48**	lh pallidum	-0.10	7.96E-04		rh inferiortemporal	-0.06	0.070	
**49**	lh pericalcarine	0.10	9.79E-04		rh parahippocampal	-0.05	0.078	
**50**	lh superiorparietal	-0.10	0.001		lh medialorbitofrontal	0.05	0.130	
**51**	lh parsorbitalis	0.09	0.002		rh pericalcarine	0.05	0.137	
**52**	rh inferiorparietal	-0.09	0.002		rh isthmuscingulate	0.04	0.182	
**53**	lh superiortemporal	0.09	0.003		lh cuneus	-0.04	0.184	
**54**	rh transversetemporal	0.09	0.004		lh isthmuscingulate	-0.04	0.206	
**55**	rh frontalpole	-0.09	0.005		rh postcentral	0.04	0.220	
**56**	lh fusiform	-0.08	0.006		lh transversetemporal	0.04	0.227	
**57**	lh rostralanteriorcingulate	-0.08	0.007		rh middletemporal	-0.04	0.247	
**58**	lh middletemporal	-0.07	0.015		lh lateralorbitofrontal	-0.03	0.297	
**59**	rh caudate	-0.07	0.024		rh insula	-0.03	0.302	
**60**	lh entorhinal	-0.06	0.040		rh bankssts	0.03	0.313	
**61**	lh cuneus	0.05	0.073		rh paracentral	-0.03	0.351	

**62**	lh parahippocampal	-0.05	0.098		lh parstriangularis	-0.03	0.390	
**63**	rh paracentral	0.05	0.101		rh entorhinal	-0.03	0.399	
**64**	rh precentral	0.05	0.116		lh rostralanteriorcingulate	-0.02	0.431	
**65**	lh isthmuscingulate	0.05	0.139		rh lingual	0.02	0.566	
**66**	lh lingual	0.04	0.163		rh frontalpole	0.02	0.585	
**67**	rh lateraloccipital	0.04	0.191		rh rostralmiddlefrontal	0.02	0.592	
**68**	lh insula	-0.04	0.198		rh medialorbitofrontal	0.01	0.746	
**69**	lh bankssts	-0.04	0.215		rh superiortemporal	0.01	0.793	
**70**	lh frontalpole	-0.04	0.220		lh paracentral	0.01	0.810	
**71**	rh pallidum	0.04	0.224		lh frontalpole	-0.01	0.836	
**72**	lh rostralmiddlefrontal	0.02	0.429		lh parsorbitalis	0.01	0.842	
**73**	rh precuneus	-0.02	0.481		rh caudalmiddlefrontal	-0.01	0.848	
**74**	lh caudalmiddlefrontal	0.02	0.535		rh fusiform	0.00	0.924	
**75**	lh lateralorbitofrontal	-0.01	0.693		rh caudate	0.00	0.933	
**76**	rh postcentral	-0.01	0.715		rh cuneus	0.00	0.972	
**77**	lh lateraloccipital	-0.01	0.849		lh precentral	0.00	0.975	
**78**	lh medialorbitofrontal	0.00	0.874		rh lateraloccipital	0.00	0.991	
**79**	rh superiorparietal	0.00	0.982		lh pericalcarine	0.00	1.000	

* p<0.01 (Bonferroni corrected for multiple comparisons)

Supplementary Table 3. Structural covariance analysis for T1w/T2w values. Pearson correlation coefficients between the claustrum and other brain regions are presented alongside the uncorrected p-values, across N=1068 subjects. Regions names are listed using their according to their absolute correlation value in descending order. Statistical significance (p<0.01) is indicated by an asterisk, after Bonferroni correction for multiple comparisons (474 comparisons: 2 hemispheres x 79 regions x 3 measures). The names of the cortical regions are listed according to the FreeSurfer labels of the Desikan-Killiany atlas.

	**Left Claustrum**				**Right Claustrum**			
	ROI	r	p		ROI	r	p	
**1**	lh putamen	0.66	0	*	rh putamen	0.64	0	*
**2**	lh thalamus proper	0.57	0	*	rh insula	0.58	0	*
**3**	rh parstriangularis	-0.56	0	*	lh putamen	-0.55	0	*
**4**	lh pallidum	0.56	0	*	lh thalamus proper	-0.52	0	*
**5**	lh posteriorcingulate	-0.55	0	*	lh lingual	-0.45	0	*
**6**	rh superiortemporal	-0.51	0	*	lh fusiform	-0.45	0	*
**7**	lh insula	0.50	0	*	lh supramarginal	0.44	0	*
**8**	lh fusiform	0.49	0	*	rh parstriangularis	0.44	0	*
**9**	rh thalamus proper	0.48	0	*	rh fusiform	0.43	0	*
**10**	lh supramarginal	-0.48	0	*	lh caudalmiddlefrontal	0.42	2.80E-45	*
**11**	lh caudalmiddlefrontal	-0.46	0	*	rh bankssts	0.42	5.61E-45	*

**12**	lh hippocampus	0.45	0	*	lh parstriangularis	-0.40	1.45E-40	*
**13**	rh rostralmiddlefrontal	-0.44	0	*	lh posteriorcingulate	0.38	7.14E-36	*
**14**	lh precuneus	-0.42	0	*	lh transversetemporal	-0.36	1.47E-31	*
**15**	rh fusiform	-0.41	1.34E-41	*	lh inferiorparietal	0.35	2.62E-31	*
**16**	rh putamen	-0.40	1.72E-39	*	lh parsopercularis	-0.31	1.75E-24	*
**17**	lh inferiorparietal	-0.39	1.12E-38	*	lh pallidum	-0.30	2.99E-22	*
**18**	rh middletemporal	-0.38	1.83E-36	*	rh rostralmiddlefrontal	0.29	6.40E-21	*
**19**	lh parstriangularis	0.36	1.69E-31	*	rh posteriorcingulate	0.26	1.12E-16	*
**20**	lh superiorfrontal	-0.35	2.36E-30	*	rh hippocampus	0.25	4.55E-16	*
**21**	rh parsorbitalis	-0.35	9.28E-30	*	lh bankssts	-0.24	3.83E-15	*
**22**	rh precuneus	-0.31	3.00E-24	*	rh superiortemporal	0.24	4.92E-15	*
**23**	rh posteriorcingulate	-0.31	3.43E-24	*	lh lateraloccipital	-0.24	6.30E-15	*
**24**	rh insula	-0.29	3.95E-21	*	lh caudate	-0.23	4.68E-14	*
**25**	lh lingual	0.29	7.55E-21	*	rh thalamus proper	-0.23	3.46E-13	*
**26**	lh cuneus	-0.28	1.89E-19	*	rh precuneus	0.22	1.24E-12	*
**27**	rh lateraloccipital	-0.27	5.41E-18	*	rh rostralanteriorcingulate	0.21	2.18E-11	*
**28**	rh entorhinal	0.26	1.34E-17	*	rh parsopercularis	0.21	2.24E-11	*
**29**	rh temporalpole	0.26	1.58E-17	*	lh precuneus	0.20	9.84E-11	*
**30**	rh frontalpole	-0.26	3.02E-17	*	rh pallidum	0.20	3.21E-10	*
**31**	rh inferiortemporal	-0.25	2.00E-15	*	lh lateralorbitofrontal	-0.19	6.66E-10	*
**32**	rh bankssts	-0.24	5.31E-15	*	lh insula	-0.19	1.71E-09	*
**33**	rh lateralorbitofrontal	-0.24	6.48E-15	*	lh isthmuscingulate	-0.19	1.78E-09	*
**34**	lh paracentral	-0.24	9.82E-15	*	rh supramarginal	-0.18	3.12E-09	*
**35**	rh parsopercularis	-0.23	5.98E-14	*	lh inferiortemporal	-0.18	6.95E-09	*
**36**	lh caudalanteriorcingulate	-0.22	1.31E-12	*	lh superiortemporal	-0.16	2.01E-07	*
**37**	rh superiorfrontal	-0.22	1.78E-12	*	rh transversetemporal	0.15	8.62E-07	*
**38**	rh caudate	0.21	7.00E-12	*	rh parahippocampal	0.15	2.91E-06	*
**39**	rh cuneus	-0.20	1.45E-10	*	lh rostralmiddlefrontal	-0.14	7.97E-06	*
**40**	lh inferiortemporal	0.19	5.69E-10	*	lh pericalcarine	-0.14	8.63E-06	*
**41**	rh superiorparietal	-0.19	6.56E-10	*	lh parahippocampal	-0.14	8.87E-06	*
**42**	lh rostralmiddlefrontal	-0.18	3.32E-09	*	lh claustrum	-0.14	9.19E-06	*
**43**	rh hippocampus	0.18	1.11E-08	*	lh superiorfrontal	0.12	1.41E-04	
**44**	rh postcentral	-0.18	1.19E-08	*	rh lingual	-0.12	1.86E-04	
**45**	rh medialorbitofrontal	-0.18	2.01E-08	*	rh paracentral	0.12	2.28E-04	
**46**	rh precentral	-0.16	1.82E-07	*	rh caudalmiddlefrontal	-0.11	3.31E-04	
**47**	lh frontalpole	-0.16	3.73E-07	*	lh cuneus	0.10	0.001	
**48**	lh transversetemporal	0.14	5.80E-06	*	rh caudate	0.10	0.001	
**49**	lh medialorbitofrontal	-0.14	7.94E-06	*	lh medialorbitofrontal	-0.10	0.002	
**50**	rh claustrum	-0.14	9.19E-06	*	rh inferiorparietal	-0.10	0.002	
**51**	lh rostralanteriorcingulate	-0.13	2.88E-05		lh middletemporal	-0.09	0.004	
**52**	rh rostralanteriorcingulate	-0.13	4.44E-05		rh middletemporal	0.09	0.005	
**53**	rh supramarginal	0.12	7.63E-05		rh pericalcarine	-0.09	0.005	
**54**	rh parahippocampal	0.12	2.00E-04		lh paracentral	0.08	0.009	

**55**	rh transversetemporal	-0.10	0.001	rh lateralorbitofrontal	0.08	0.010
**56**	lh parsorbitalis	0.10	0.001	lh postcentral	-0.08	0.011
**57**	rh isthmuscingulate	0.09	0.002	rh isthmuscingulate	-0.08	0.016
**58**	rh lingual	0.09	0.003	rh parsorbitalis	0.07	0.018
**59**	lh caudate	0.08	0.008	rh postcentral	0.07	0.029
**60**	lh parahippocampal	0.08	0.014	rh frontalpole	0.07	0.031
**61**	lh isthmuscingulate	-0.07	0.023	rh entorhinal	-0.07	0.032
**62**	lh middletemporal	0.07	0.023	rh precentral	-0.06	0.042
**63**	lh superiortemporal	0.06	0.077	lh precentral	0.06	0.062
**64**	rh caudalanteriorcingulate	-0.05	0.081	lh entorhinal	0.05	0.149
**65**	rh pallidum	-0.05	0.146	lh parsorbitalis	-0.04	0.167
**66**	lh lateraloccipital	0.04	0.197	lh frontalpole	-0.04	0.167
**67**	rh caudalmiddlefrontal	-0.04	0.205	lh temporalpole	0.04	0.264
**68**	lh superiorparietal	0.04	0.207	lh rostralanteriorcingulate	0.03	0.269
**69**	rh inferiorparietal	0.04	0.239	rh superiorfrontal	0.03	0.280
**70**	lh temporalpole	0.04	0.241	rh medialorbitofrontal	-0.03	0.283
**71**	lh bankssts	-0.04	0.256	rh cuneus	0.03	0.321
**72**	lh precentral	-0.03	0.302	rh temporalpole	-0.03	0.342
**73**	lh lateralorbitofrontal	0.03	0.331	rh caudalanteriorcingulate	-0.03	0.349
**74**	lh entorhinal	0.02	0.495	lh superiorparietal	0.03	0.391
**75**	rh pericalcarine	-0.02	0.591	rh inferiortemporal	0.02	0.515
**76**	lh parsopercularis	0.02	0.617	rh superiorparietal	-0.02	0.620
**77**	lh postcentral	-0.01	0.678	lh hippocampus	0.01	0.646
**78**	rh paracentral	-0.01	0.750	rh lateraloccipital	-0.01	0.767
**79**	lh pericalcarine	-0.01	0.777	lh caudalanteriorcingulate	0.00	0.981

* $p<0.01$ (Bonferroni corrected for multiple comparisons)

Supplementary Table 4. Structural covariance analysis for mean diffusivity (MD). Pearson correlation coefficients between the claustrum and other brain regions are presented alongside the uncorrected p-values, across N=1014 subjects. Regions names are listed using their according to their absolute correlation value in descending order. Statistical significance ($p<0.01$) is indicated by an asterisk, after Bonferroni correction for multiple comparisons (474 comparisons: 2 hemispheres x 79 regions x 3 measures). The names of the cortical regions are listed according to the FreeSurfer labels of the Desikan-Killiany atlas.
